# Bacillus-infecting bacteriophage Izhevsk harbors thermostable endolysin with broad range specificity

**DOI:** 10.1371/journal.pone.0242657

**Published:** 2020-11-24

**Authors:** Anna V. Skorynina, Emma G. Piligrimova, Olesya A. Kazantseva, Vladislav A. Kulyabin, Svetlana D. Baicher, Natalya A. Ryabova, Andrey M. Shadrin

**Affiliations:** 1 G.K. Skryabin Institute of Biochemistry and Physiology of Microorganisms, Pushchino Scientific Center for Biological Research of the Russian Academy of Sciences, Federal Research Center, Pushchino, Russia; 2 Institute of Protein Research RAS, Pushchino, Russia; Roskilde Universitet, DENMARK

## Abstract

Several bacterial species belonging to the *Bacillus cereus* group are known to be causative agents of food poisoning and severe human diseases. Bacteriophages and their lytic enzymes called endolysins have been widely shown to provide for a supplemental or primary means of treating bacterial infections. In this work we present a new broad-host-range phage Izhevsk, which infects the members of the *Bacillus cereus* group. Transmission electron microscopy, genome sequencing and comparative analyses revealed that Izhevsk is a temperate phage with *Siphoviridae* morphology and belongs to the same genus as the previously described but taxonomically unclassified bacteriophages Tsamsa and Diildio. The Ply57 endolysin of Izhevsk phage has broad-spectrum activity against *B*. *cereus sensu lato*. The thermolability of Ply57 is higher than that of the PlyG of Wβ phage. This work contributes to our current understanding of phage biodiversity and may be useful for further development of efficient antimicrobials aimed at diagnosing and treating infectious diseases and food contaminations caused by the *Bacillus cereus* group of bacteria.

## Introduction

In the early 2000s, the growing availability of sequencing technologies and the increasing emergence rate of multi-resistant pathogens served as the main factors which triggered a revived interest in phage research worldwide. Bacteriophages and their constituents are now widely used in medical diagnostics and food microbiology, and their applicability continues to expand in the treatment of human infections. Despite being undeniably limited compared to conventional antibacterial therapies, they are gaining more and more recognition. Many reports have been published describing phages and their bacteriolytic enzymes–natural endolysins or engineered derivatives–successfully used to prevent contamination of food products [1,2], as well as investigated in rodent-based models of human infections [3,4]. Endolysins are phage-encoded peptidoglycan-degrading proteins required for host cell wall lysis and possessing different enzymatic activities such as glycosidase, amidase, endopeptidase and lytic transglycosylase [5]. In the case of phages infecting Gram-positive bacteria, endolysins consist of one or more catalytic domains and a cell wall binding domain [5,6], offering wide opportunities for combining different molecules and generating proteins targeted to specific substrates.

*Bacillus cereus sensu stricto* is a Gram-positive spore-forming bacterium widespread in various environmental niches. It is also a member of the *Bacillus cereus* group of bacteria encompassing 18 recognized species [7] including *Bacillus anthracis*, a severe human pathogen. Many reports have shown that the members of the *Bacillus cereus* group commonly contaminate milk and dairy products [8–10], presenting potential threats because of their ability to grow at standard refrigerator temperatures and produce toxins and spores. Bacteriophages infecting bacteria of the *Bacillus cereus* group were among the first to be detected. Long before the term “bacteriophage” was proposed by D’Herelle, Nikolai Fedorovich Gamaleya observed antibacterial activity while working with *Bacillus anthracis* [11].

As one may notice from the information given in the “Taxonomy” section on the International Committee on the Taxonomy of Viruses (ICTV) website, *Bacillus*-infecting phages are now among the most numerous taxonomically classified bacteriophages of the order *Caudovirales* together with the viruses infecting *Enterobacteriaceae*, *Mycobacterium* and *Staphylococcus*. They constitute many genera of the *Myoviridae*, *Siphoviridae*, *Podoviridae* and *Herelleviridae* families and are also represented by a considerable number of taxonomically unclassified phages deposited in public databases.

The impressive variety of morphological features, genome characteristics, lifestyles, and lysogenic strategies have been reported for *Bacillus*-infecting bacteriophages [12], indicating that they are widespread, diverse and therefore of significant interest.

In this study, we describe the newly isolated temperate phage Izhevsk and characterize its thermostable broad-spectrum endolysin Ply57. The analysis of the Izhevsk genome has shown that the phage can be assigned to the same genus with the previously described and closely related *Bacillus*-infecting phages Tsamsa and Diildio, but the lytic range of Izhevsk phage is much wider than that of the previously described phages.

## Materials and methods

### Bacterial strains and growth conditions

Bacterial strains were obtained from the All-Russian Collection of Microorganisms (VKM) and cultivated in Lysogeny broth (LB) and on LB agar (1.5% and 0.5% for top layer) with 10 mM of CaCl_2_ and 10 mM of MgCl_2_ at 30°C.

### Phage isolation and propagation

Phage Izhevsk was isolated from a soil sample collected in Izhevsk, Udmurtia, Russian Federation, and propagated on the sensitive strain *B*. *cereus* VKM B-370. For phage isolation, one gram of the soil sample was added to the cell culture at the optical density (OD590) of 0.2 and the mixture was incubated until the optical density decreased. The lysate was filtered through 0.22-μm filters.

Serial dilutions of the phage suspension were prepared and added to 3 ml of 0.5% LB agar with 50 μL of the *B*. *cereus* VKM B-370 culture (OD590 of 0.35); then, after 15 sec vortexing, the mixture was poured into previously prepared LB agar plates (1.5% agar) and incubated overnight at 37°C.

Phage purification, propagation and PEG 8000 (polyethylene glycol) precipitation were performed as described previously [13]. Then 3 ml of the high-titer preparation were used for CsCl centrifugation in a preformed CsCl density gradient (1.3 g/ml, 1.4 g/ml, 1.5 g/ml, 1.6 g/ml, 1.7 g/ml) to prepare the final purified phage suspension, which was used later on for transmission electron microscopy.

### Phage DNA sequencing

Phage DNA was extracted using the standard phenol-chloroform extraction protocol described by Sambrook et al. [14] and then sequenced using Illumina with the TruSeq library preparation technology. The genomic sequence was assembled *de novo* using SPAdes v.3.11.1 software [15]. Open reading frames (ORFs) were identified with RASTtk [16] and manually assigned the putative functions using BLAST (NCBI) [17,18] and HHpred [19]. tRNAscan-SE [20] were used for tRNA gene searching. The circular genome map was visualized with CGView [21].

### Host range determination

Host range test was performed as described previously [13] using 34 strains of the *B*. *cereus* group as well as five other *Bacillus* strains and one *Enterococcus* strain. 2 μL of phage suspension (≥10^6^ p.f.u./mL) was dripped onto agar plate with each tested strain. Plates were incubated at 37°C for 48h. Lysis was monitored after 16, 30 and 48 h of incubation.

### Transmission electron microscopy

Phage suspension applied onto 400 mesh carbon-formvar coated copper grids was negatively stained with 1% uranyl acetate and subsequently analyzed using a JEM-100С (JEOL, Japan) transmission electron microscope at 80 kV accelerating voltage. Images were taken on Kodak film SO-163 (Kodak, Cat. #74144, Hatfield, PA, USA) with 45000x magnification.

### Endolysin gene cloning and enzyme purification

The genes of the Ply57 and PlyG endolysins were PCR-amplified using Herculase II Fusion DNA Polymerase (Agilent Technologies, Cat. # 600677–51) and the pairs of oligonucleotides 5’-tatatccatggCTATTTCAGTAAGACAAAAA-3’ plus 5’-tatagcggccgcGTCTTGTACGAAACGCAC-3’ and 5’-tatatccatggAAATTCAGAAAAAATTAGTTGACCCA-3’ plus 5’-tatagcggccgcTTTAACTTCATACCACCA-3’, respectively. Total DNA of *B*. *cereus* W-strain, harboring Wβ prophage, was used as a template for PlyG cloning. The PCR products were cloned into the pET33 expression vector between the NcoI and EagI sites resulting in the AAALEHHHHHH amino acid extension at the C-terminus of the recombinant proteins. For Ply57 and PlyG production, *E*. *coli* BL21(DE3) transformed with the respective pET33-based plasmid was grown on an orbital shaking platform at 37°C up to OD590 = 0.5 and then 50 μM IPTG was added to induce recombinant protein synthesis. The temperature was changed to 30°C and the incubation continued for the next 12 hours. Bacterial cells were precipitated by centrifugation and resuspended in 40 mL of ice-cold Buffer A [40 mM Tris-HCl рН 8.0; 0.5 M NaCl; 5% glycerol]. The cell suspension was sonicated thrice for 30 seconds using a Sonyprep disintegrator (Amplitude 4 and medium power). The sonicated cells were centrifuged for 90 min at 9000 g, 4°C, filtered through 0.45 μm Millipore syringe filter, and loaded on a 5 mL Ni-chelating column (GE Healthcare). Purification was performed using imidazole elution as recommended by the manufacturer. The purification quality was confirmed by SDS polyacrylamide gel electrophoresis and the selected fractions were dialyzed against the dialysis buffer [40 mM Tris-HCl (рН 8.0); 0.5 M NaCl; 5% glycerol], which was refreshed three times. After the dialysis, protein concentrations were determined by measuring adsorption at λ = 280 nm (extinction coefficient = 1) using NanoPhotometer Pearl P-360, and then glycerol and DTT were added to the final concentrations of 50% and 10 mM, respectively. The enzymes were stored at -20°C. The final concentration of the purified enzymes was 100–150 μM.

### Endolysin specificity spot test

5 μl of Ply57 (100 μM) was dripped onto Brain Heart Infusion (BHI) agar plates with 4-hour lawns of each host strain, the Ply57 storage buffer was used as a control. The plates were incubated at 37°C for 16–48 hours. When a lysis zone was detected after incubation, host strain was considered sensitive.

### Endolysin activity turbidimetry assays

The bacteriolytic activity of the purified Ply57 endolysin was determined via turbidity reduction assay on fresh exponentially growing bacterial cells as described previously [22], as well as on stationary phase cells, with minor modifications. Bacterial cells were precipitated, resuspended in 20 mM Tris-HCl (pH 8.0) buffer to the OD590 of approximately 1.0, and aliquoted into a 96-well plate (190 μl per each well). The purified endolysin was diluted in 20 mM Tris-HCl buffer to obtain 20 μM working solution. Ten microliters of the working solution were added to the bacterial cells resulting in the final volume of 200 μl with 1 μM of endolysin. The same amount of storage buffer was added to the control microplate well without endolysin. The plate was incubated at 37°C in the microplate spectrophotometer FilterMax F5 (Molecular Devices) with OD590 being measured every minute. The tested strains were considered sensitive during the exponential growth when OD590 decreased to the values lower than 80% of the control optical density (in the cultures without endolysin) after 30-min incubation. The strains were considered sensitive during the stationary phase when OD590 decreased to the values lower than 80% of the control optical density (in the cultures without endolysin) after 1 h incubation. Each experiment was performed in at least three replicates.

Thermostability assay was performed as described earlier by Heselpoth et al. [23] with several modifications. Ply57 and PlyG were incubated at 55°C in PBS (40 mM sodium phosphate pH 7.4; 150 mM NaCl) without reducing agents. Aliquots were taken after 10, 30, 60, 90 and 120 min and placed on ice bath until 120 min incubation was complete. The residual bacteriolytic activity was determined on exponentially growing *B*. *cereus* ATCC 4342 (also known as RSVF1) cells in PBS using the microplate spectrophotometer. The final concentrations of Ply57 and PlyG in the reactions were 1.5 μM and the reaction mixture volume was 200 μl. The endolysin half-inactivation time was estimated using the exponential trendline equation for the dependence of the residual bacteriolytic activity from incubation time (R^2^ value >0.98). The average values and standard deviations were calculated based on at least three replicates.

### Comparative genomics

To find the related phages, we performed a BLASTN search using the whole genome sequence of Izhevsk as the query. The linear comparison diagram showing the similarity between phage genomes was produced with EasyFig [24]. The number of proteins shared by phages was computed using the GET_HOMOLOGUES software [16] with the COGtriangles algorithm [17,18] (-t 0 –C 50 -e). VICTOR [25] was used for phylogenetic inference from the whole proteome sequences of Izhevsk and the closest viruses using the Genome-BLAST Distance Phylogeny method [26] with the settings recommended for prokaryotic viruses [25]. The resulting tree was rooted at the midpoint [27] and visualized with FigTree v1.4.4 [28].

### Accession number

The genome sequence of phage Izhevsk is available in the GenBank under accession number MT254578.

## Results

### Isolation, host range and lytic spectrum

Phage Izhevsk was isolated from soil samples collected in Izhevsk, Udmurtia, Russian Federation, in 2016. The host range was determined on 34 bacterial strains of 3 different species belonging to the *Bacillus cereus sensu lato* group (Table 1). The phage was capable of forming plaques on the lawns of 33 strains. On the propagating host strain *B*. *cereus* VKM B-370, Izhevsk produced turbid plaques with an approximate diameter of 1 mm (S1 Fig).

**Table 1:** The host range of phage Izhevsk and the lytic spectrum of its endolysin determined on 34 different *Bacillus cereus sensu lato* strains.

No.	Species	Strain	Phage Lysis	Endolysin lysis		
				Exp. phase*	Stat. phase	Spot test
1	*B*. *cereus*	VKM B-13	+	+++	+	+
2	*B*. *cereus*	VKM B-370	+	+++	-	+
3	*B*. *cereus*	VKM B-373	+	+++	-	+
4	*B*. *cereus*	VKM B-374	+	+++	+	+
5	*B*. *cereus*	VKM B-383	+	-	+	+
6	*B*. *cereus*	VKM B-445	+	++	+	+
7	*B*. *cereus*	VKM B-473	+	+	-	+
8	*B*. *cereus*	VKM B-491	+	+++	+	+
9	*B*. *cereus*	VKM B-504^T^	+	++	+	+
10	*B*. *cereus*	VKM B-681	+	++	+	+
11	*B*. *cereus*	VKM B-682	+	+++	+	+
12	*B*. *cereus*	VKM B-683	+	+++	+	+
13	*B*. *cereus*	VKM B-684	+	+++	+	+
14	*B*. *cereus*	VKM B-686	+	+++	+	+
15	*B*. *cereus*	VKM B-688	+	+	+	+
16	*B*. *cereus*	VKM B-771	+	+	+	+
17	*B*. *cereus*	VKM B-810	+	++	+	+
18	*B*. *cereus*	VKM B-812	-	++	+	+
19	*B*. *cereus*	ATCC 4342	+	+++	+	+
20	*B*. *cereus*	ATCC 14893	+	++	+	-
21	*B*. *thuringiensis*	VKM B-83	+	+++	+	+
22	*B*. *thuringiensis*	VKM B-84	+	+++	+	+
23	*B*. *thuringiensis*	VKM B-85	+	+++	+	+
24	*B*. *thuringiensis*	VKM B-440	+	-	-	+
25	*B*. *thuringiensis*	VKM B-443	+	++	+	+
26	*B*. *thuringiensis*	VKM B-446	+	++	+	+
27	*B*. *thuringiensis*	VKM B-447	+	+++	+	+
28	*B*. *thuringiensis*	VKM B-450	+	-	-	+
29	*B*. *thuringiensis*	VKM B-453	+	-	-	+
30	*B*. *thuringiensis*	VKM B-454	+	-	-	+
31	*B*. *thuringiensis*	VKM B-1555	+	+	+	+
32	*B*. *thuringiensis*	VKM B-1557	+	++	+	+
33	*B*. *thuringiensis*	ATCC 35646	+	-	+	+
34	*B*. *weihenstephanensis*	KBAB4	+	+++	+	+
	*B*. *flexus*		-	++	+	-
	*B*. *pumilus*		-	-	-	-
	*B*. *subtilis*	168	-	+	-	+
	*B*. *subtilis*	WB 800n	-	-	-	-
	*B*. *megaterium*	MS941	-	++	+	+
	*E*. *faecium*	FS86	-	-	-	-

* Results recorded as “+++”, complete or near to complete lysis; “++”, moderate lysis; “+”, slight lysis; and “−“, no detectable lysis.

### Transmission electron microscopy

**Fig 1:**
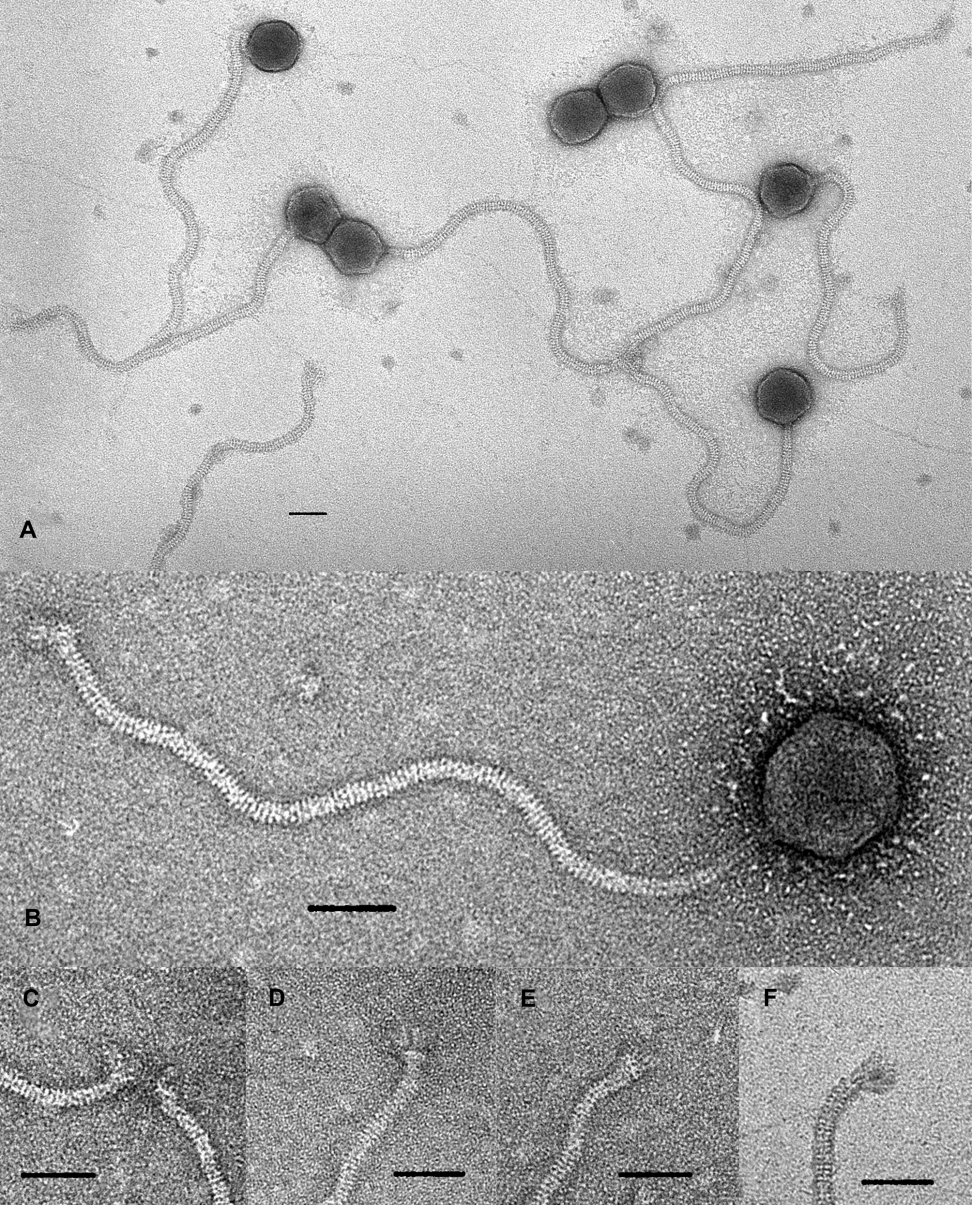
Transmission electron microscopy of *Bacillus* phage Izhevsk particles negatively stained with 1% (w/v) uranyl acetate. Scale bars are 50 nm. А. The bacteriophage particles. B. Closeup view of a single phage particle. C-F. Baseplate and fiber-like structures, closeup view.

TEM analysis revealed that Izhevsk possesses a long flexible non-contractile tail with an approximate length of 430 nm and a non-elongated capsid, approximately 80 nm in diameter (Fig 1), the two features characteristic of the *Siphoviridae* B1 morphotype [29]. The tail contains about 120 visible striations (disk-like structures) (Fig 1B). At the end of the tail, the baseplate structure is visible with extending fiber-like structures of up to 30 nm (Fig 1C–1F), through which some phage particles are attached to one another (Fig 1A–1C).

### Genome organization

**Fig 2:**
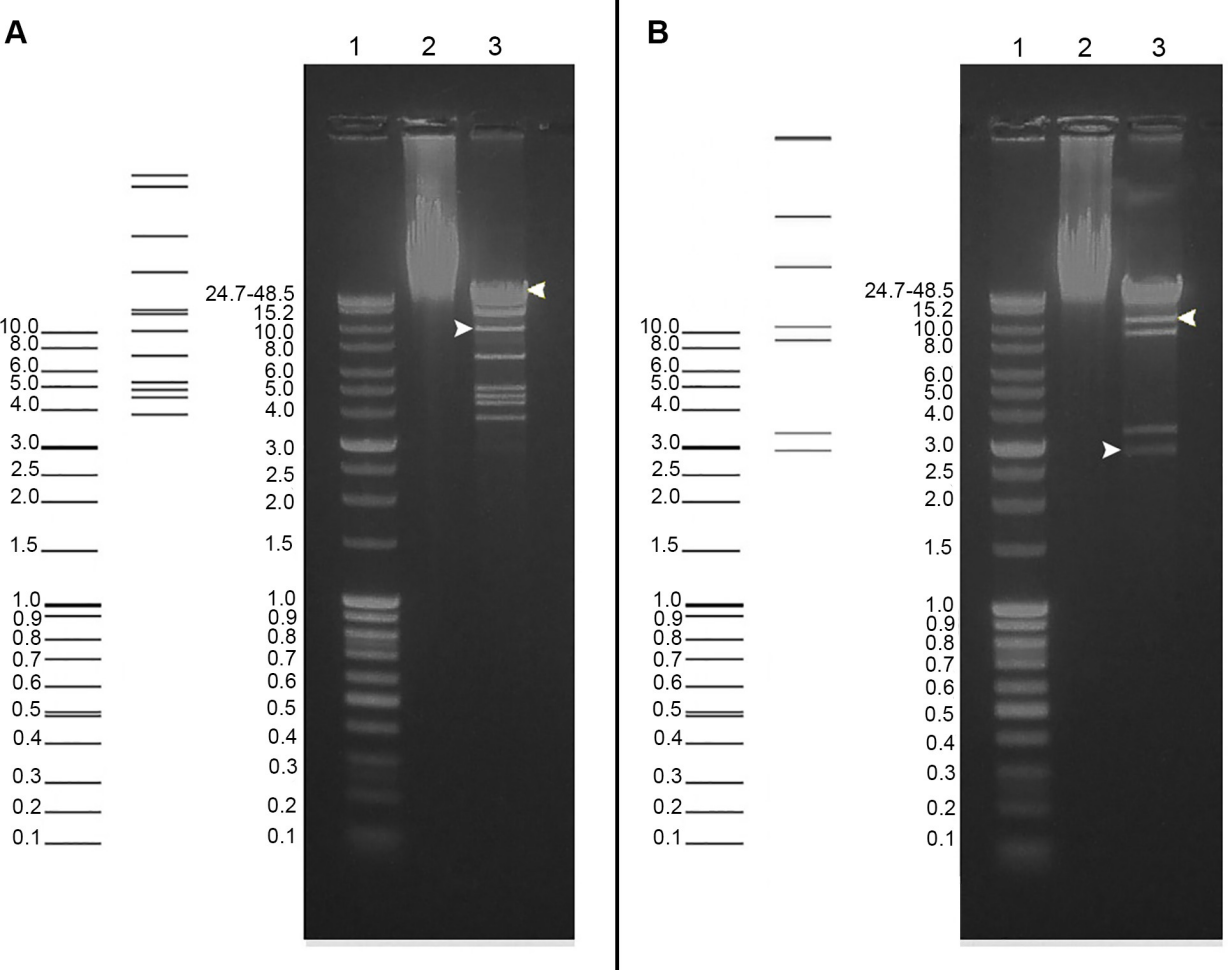
Phage DNA digested with restriction endonucleases PacI (A) and AfeI (B) *in silico* and *in vitro* (the left and right side of each image, respectively). Lanes (for each image): 1 –molecular weight markers (SibEnzyme, mix of Cat.# M29 and M33), 2 –intact phage DNA, 3 –digested DNA. The lengths of the fragments predicted for PacI: 33909, 32075, 24289, 18753, 13056, 12356, 9779, 6893, 4963, 4570, 4274, 3721; for AfeI: 54176, 40885, 27581, 19892, 10860, 8996, 3337, 2920. Terminal fragments are underlined here in the text and indicated by the arrows on the electrophoregrams.

A single circular contig with the length of 168350 bp, the average coverage of 68x and the GC-content of 34.3% was generated from sequencing reads. A simple commonly used method to predict phage genome termini is to find coverage deviations when aligning reads on the assembled genomic sequence [30]. No regions deviating ≥2 times from the average coverage were presented on read coverage diagram (not shown), so it was complicated to find the genome termini by this approach. The putative ends positions were roughly located taking into account the significant similarity between the Izhevsk genomic sequence and that of the previously described bacteriophage vB_BanS_Tsamsa with 284-bp DTR regions determined by the authors [31]. The postulate was then proven by electrophoresis of the Izhevsk DNA digested with the PacI and AfeI restriction enzymes (Fig 2).

**Fig 3:**
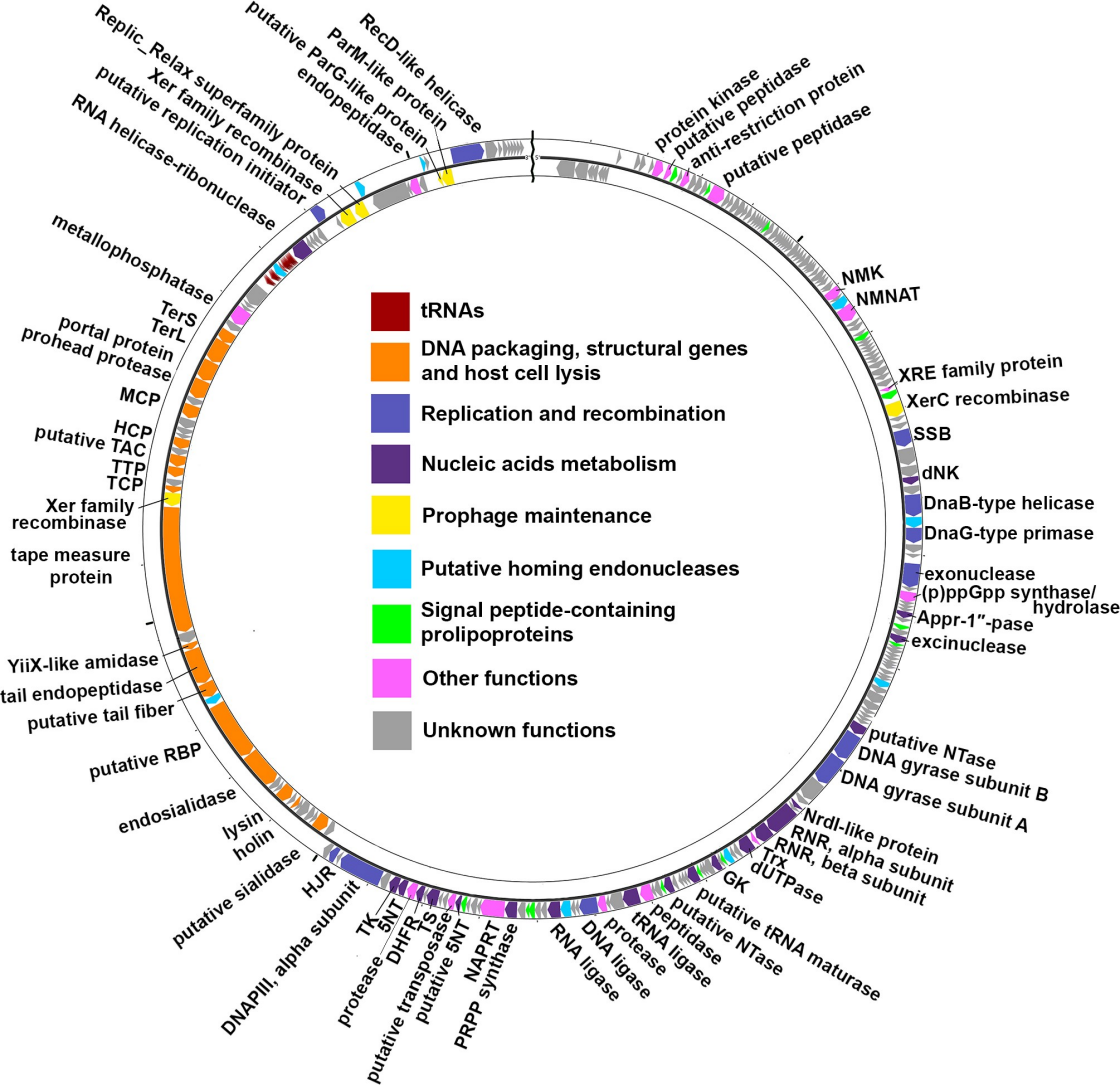
The Izhevsk genome map. Functionally assigned ORFs are highlighted according to their general functions (see the legend). Abbreviations: NMK–nicotinamide mononucleotide transporter, NMNAT—nicotinamide mononucleotide adenylyltransferase, SSB–single-stranded DNA-binding protein, dNK—deoxyribonucleoside kinase, NTase–nucleotidyltransferase, RNR—Ribonucleotide reductase, Trx–thioredoxin, GK—guanylate kinase, PRPP synthase—Phosphoribosyl diphosphate synthase, NAPRT—Nicotinate Phosphoribosyltransferase, 5NT—5′-Nucleotidase, TS—Thymidylate synthase, DHFR—Dihydrofolate reductase, TK—thymidine kinase, DNAP–DNA polymerase, HJR–Holliday junction resolvase, RBP–receptor-binding protein, TCP–tail completion protein, TTP–tail tube protein, TAC–tail assembly chaperone, HCP–head completion protein, MCP–major capsid protein, TerL–terminase, large subunit, TerS–terminase, small subunit.

The genome contains 256 predicted protein-encoding ORFs; 80 of them (31.25%) were functionally assigned. Like many phages with large genomes, the Izhevsk genes can be classified into several functional groups (Fig 3), with most of them scattered over more than half of the genome and not necessarily forming visible ‘gene modules’ characteristic of phages with smaller genomes.

Among the best-annotated groups of DNA packaging, structural and host lysis-related genes, we have identified those encoding small and large terminase subunits, portal protein, putative prohead protease, major capsid protein, head completion protein, putative assembly chaperone, tail tube protein, tail completion protein, tape measure protein, YiiX-like amidase, putative tail endopeptidase, putative tail fiber protein, putative receptor-binding protein, endosialidase, holin and endolysin with the N-acetylmuramoyl-L-alanine amidase activity.

Within the cluster of tail genes and in other parts of the genome, we have identified three genes (ORFs 208, 237, 80) encoding the Xer family recombinases known to participate in resolving the dimeric forms of circular DNA molecules such as bacterial chromosomes and plasmids [32]. There have also been several reports on the ability of these proteins to enable integration of mobile elements into the *Vibrio cholerae* chromosome [33–35]. Three of the genes located near ORF237 encode the Replic_Relax superfamily protein (ORF238), ParM-like protein (ORF247) and putative ParG-like protein (ORF246), the common components of plasmid relaxation and partitioning systems. Temperate *Bacillus* phages possess diverse lifestyles and can integrate their DNA into host chromosomes and plasmids or replicate without integration as circular or linear plasmids [12]. Phages with circular plasmid lysogenic stages usually feature the Xer family recombinases and ParMRC or similar segregation systems [36,37]. Thus, taking into account the presence of plasmid replication and plasmid segregation-related set of genes, it seems possible that Izhevsk can exist as a circular plasmid prophage in the host cytoplasm while not necessarily integrating its DNA into the host chromosome.

The replication and recombination-related genes of Izhevsk encode the putative lambda O-type replication initiator, RecD-like DNA helicase, single-stranded DNA-binding protein, DnaB-type helicase, DnaG-type primase, RecJ-type DNA exonuclease, DNA gyrase subunit A, DNA gyrase subunit B, DNA ligase, DNA polymerase III alpha subunit and RuvC-type Holliday junction resolvase.

Another unusually large gene group highlighted in purple on the genome map includes the genes encoding products involved in different processes of nucleic acid metabolism such as DNA nucleotide synthesis, repair and tRNA splicing: deoxyribonucleoside kinase, Appr-1”p-processing protein, excision endonuclease, putative nucleotidyltransferases, NrdI-like protein, alpha and beta subunits of ribonucleotide reductase, dUTP diphosphatase, guanylate kinase, putative tRNA maturase, tRNA ligase, RNA ligase, phosphoribosyl pyrophosphate synthetase, putative 5’-nucleotidases, thymidine kinase and RNA helicase-ribonuclease.

**Fig 4:**
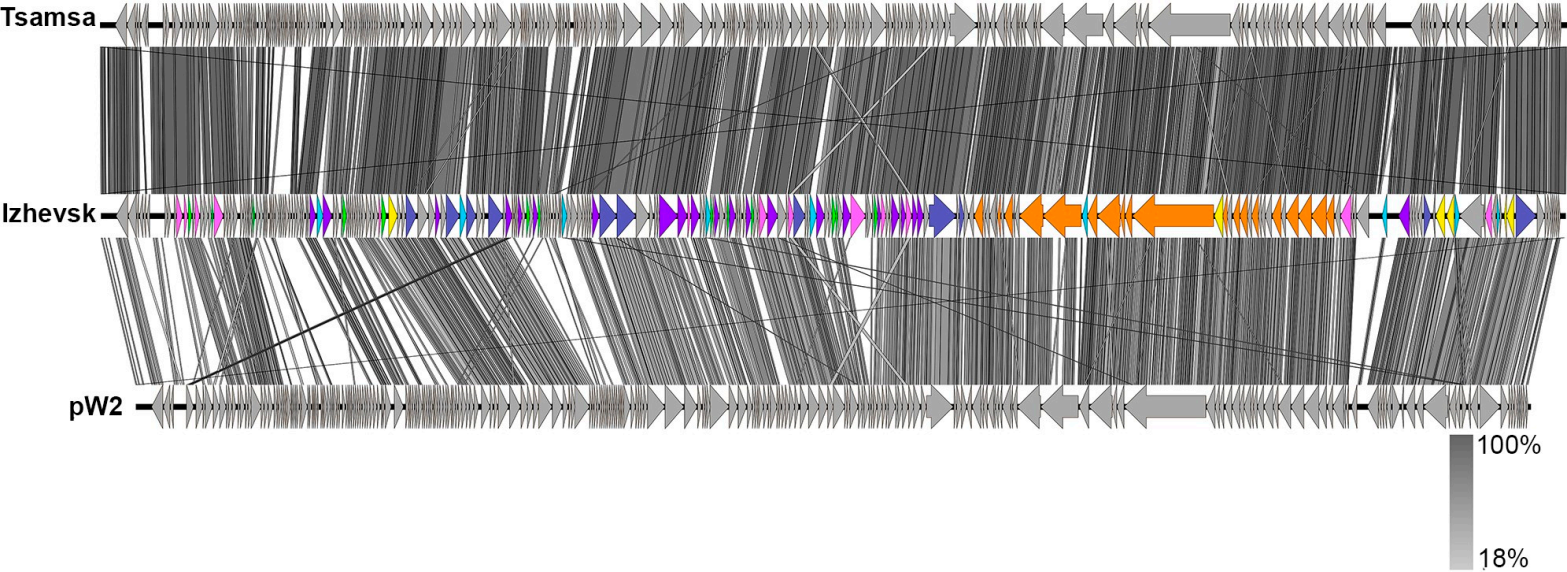
Pairwise TBLASTX comparisons of the Izhevsk genome and the related genomes visualized with Easyfig. The Izhevsk genome scheme coloring corresponds to Fig 3. Grey areas between the genome maps indicate the level of identity (see the legend).

In total, nine genes were predicted to encode HNH- and GIY-YIG-family endonucleases and were assigned the putative homing endonuclease function based on their absence in the respective positions in the genomes of related phages, Tsamsa and pW2 (Fig 4), which may be considered evidence of their potential mobility [38]. Homing endonucleases are mobile elements which are common in bacterial and large phage genomes and promote their own spread between related genomes by the double-strand break repair pathway [39]. All the putative homing endonucleases of Izhevsk are free-standing, although several phages possess intron-encoded homing endonucleases [40,41].

Among the rest of the genes not assigned to any of the main modules, the most noticeable is ORF17 encoding putative DNA restriction enzyme inhibitor similar to T7 protein Ocr, mimicking B-form DNA and inhibiting the restriction-modification activity of the Type I EcoKI complex [42]. Also, the NAD metabolism-related genes have been identified encoding: PnuC-like nicotinamide mononucleotide transporter (ORF59), NadR-type nicotinamide-nucleotide adenylyltransferase (ORF62) and nicotinate phosphoribosyltransferase (ORF169). In addition to the extended gene baggage that seems to include not only the minimum set needed for phage functions but also the genes beneficial for the host metabolism, Izhevsk possesses a tRNA array consisting of 16 tRNA and 2 pseudo-tRNA genes, predicted with tRNAscan-SE [20].

Altogether 13 ORFs were predicted to encode lipoprotein signal peptides. Although first described long ago [43], phage-encoded lipoproteins are still considered rare, probably because signal sequence-prediction tools are not generally included in the annotation pipelines for bacteriophage genomes. The presence of prolipoprotein-encoding ORFs is mostly characteristic of temperate phages, where these molecules have been shown to contribute to superinfection exclusion, lysis, host virulence [44] and lysis-lysogeny switch [45].

The related phage genomes were found by BLASTN search with the whole genome sequence of Izhevsk against the NCBI nr database (taxid:10239) and the *Bacillus* Phage Database (BPD) (http://bacillus.phagesdb.org/). Eight complete phage genomes, including previously described phages Tsamsa [31] and PBC2 [22], were downloaded from GenBank and another closely related phage named Diildio was downloaded from BPD.

**Fig 5:**
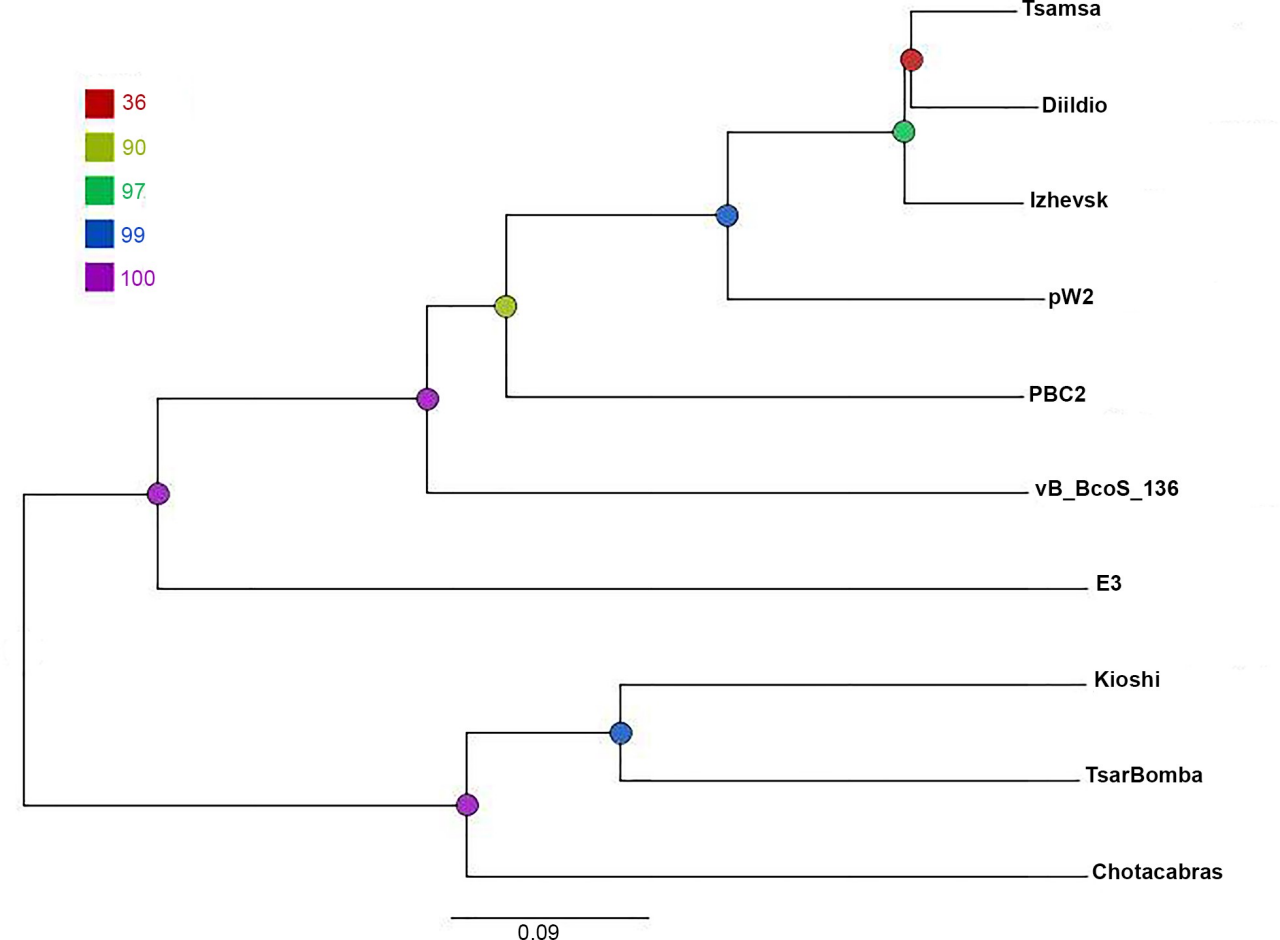
Genome-BLAST Distance Phylogeny (GBDP) phylogram based on the amino-acid sequences of whole proteomes and inferred using the formula D6. The nodes are colored according to the legend representing GBDP pseudo-bootstrap support values from 100 replications. The branch lengths are scaled in terms of the GBDP distance formula D6.

Whole-genome comparison based on the translated sequences of all predicted ORFs was performed with VICTOR (formula D6), and the resulting phylogram shown in Fig 5 illustrates the evolutionary relationship between Izhevsk and the closest viruses. The branching order is consistent with the percentile amount of proteins shared by the five most closely related phages with Izhevsk (Table 2).

**Table 2:** The properties of Izhevsk and the most closely related genomes.

Name	Database accession number	Genome length	ORFs (protein-coding	GC-content	BLASTN nucleotide identity to Izhevsk, %*	Proteins shared with Izhevsk, number (%)**	Number of tRNAs	Number of proteins shared with the phage by all of the previous phages of the table.
Izhevsk	MT254578.1	168638	256	34.3			18	
Tsamsa	NC_023007.1	168876	272	34.3	88.6	220 (80.88)	19	220
Diildio		171148	267	34.3	87.5	214 (80,15)	18	204
pW2	MK288021.1	160627	255	32.5	57.2	154 (60.39)	4	150
PBC2	KT070867.1	168689	251	34.4	42.6	128 (51)	17	103
vB_BcoS_136	MH884508.1	160590	239	32.2	29.2	101 (42,26)	17	82

*Determined using BLASTN compared to Izh57 (multiplying % coverage by % identity)

**Determined using GET_HOMOLOGUES (COGtriangles algorithm, -t 0, -C 50, -e).

### Specificity range, homologs and thermostability of Ply57

**Fig 6:**
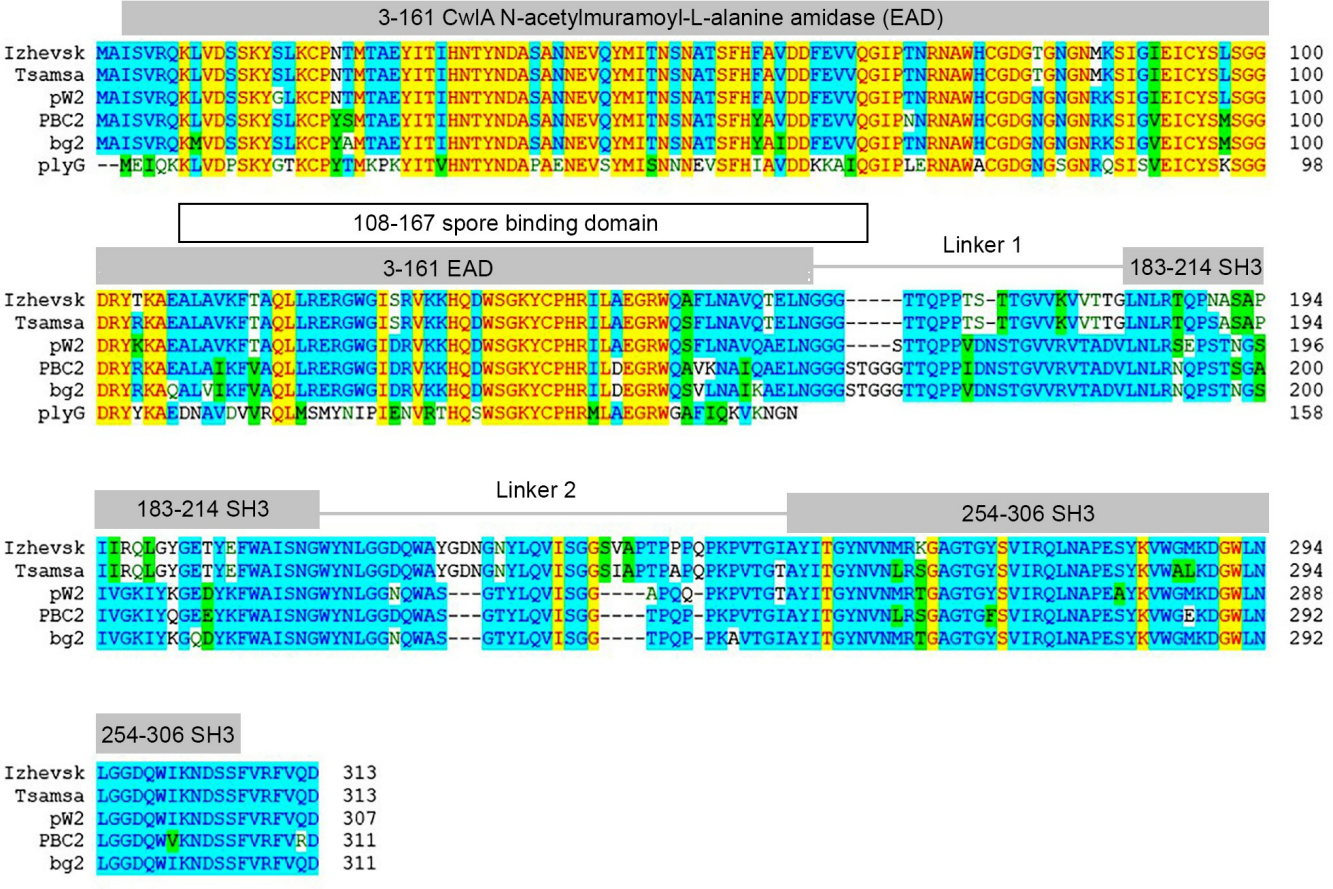
Multiple amino acid sequence alignment of Ply57 (ORF196) from bacteriophage Izhevsk with endolysins from vB_BanS-Tsamsa (YP_008873459.1), pW2 (AZU98917.1), PBC2 (AKQ08512.1), bg2 (ABX56142.1) and the enzymatically active domain of PlyG from *Bacillus* phage gamma (2L47_A). Conservative amino acids are highlighted in blue; amino acids matching the residues of the PlyG endolysin are yellow; synonymous substitutions are green. Conservative domain regions revealed with DELTA-BLAST are indicated above the aligned sequences.

ORF194 and ORF196 of phage Izhevsk were annotated as holin and endolysin, respectively. Like most endolysins from the phages infecting Gram-positive bacteria, Ply57 has a modular organization and consists of three domains: N-terminal enzymatic active domain (EAD) and two C-terminal cell wall binding domains (CBD). BLASTp analysis revealed several highly related endolysins including those of phages vB_BanS-Tsamsa (96.81% aa identity), pW2 (84.76%), PBC2 (80.88%), and bg2 (79.94%) (Fig 6). In contrast to endolysins of PBC2 and bg2 phages, Ply57, like endolysins of Tsamsa and pW2 phages, contains a short linker part connecting the EAD and CBD domains.

Like endolysins of closely related phages Tsamsa [31] and PBC2 [22], Ply57 shows a broad range of bacteriolytic activity against the collection of laboratory strains. Taking together the results of turbidimetry reduction and spot test assays, all of the 34 *B*. *cereus* group strains, as well as *B*. *megaterium* MS941, *B*. *subtilis* 168 and *B*. *flexus* IPP1, are sensitive to Ply57 (Table 1). Turbidimetry reduction assay has shown that two strains (*B*. *cereus* VKM B-383 and ATCC 35646) are not sensitive to Ply57 in the exponential growth phase, but become sensitive to Ply57 in the stationary phase. *B*. *cereus* VKM B-370, VKM B-373 and VKM B-473 strains are sensitive to Ply57 during exponential growth, but resistant in the stationary phase. *B*. *thuringiensis* VKM B-440, VKM B-453, VKM B-454 strains are not sensitive in both exponential and stationary growth phases. However, all of the nine strains mentioned above were sensitive to Ply57 in spot assay. On the other hand, *B*. *cereus* ATCC 14893 was not sensitive to Ply57 in spot test, but appeared to be sensitive in turbidimetry reduction assay (Table 1). Apparently, the bacteriolytic spectrum of endolysins must be tested using several different approaches to exclude false-negative results, which may be due to changes in the peptidoglycan structure during bacterial culture growth as well as changes in growth conditions [46].

**Fig 7:**
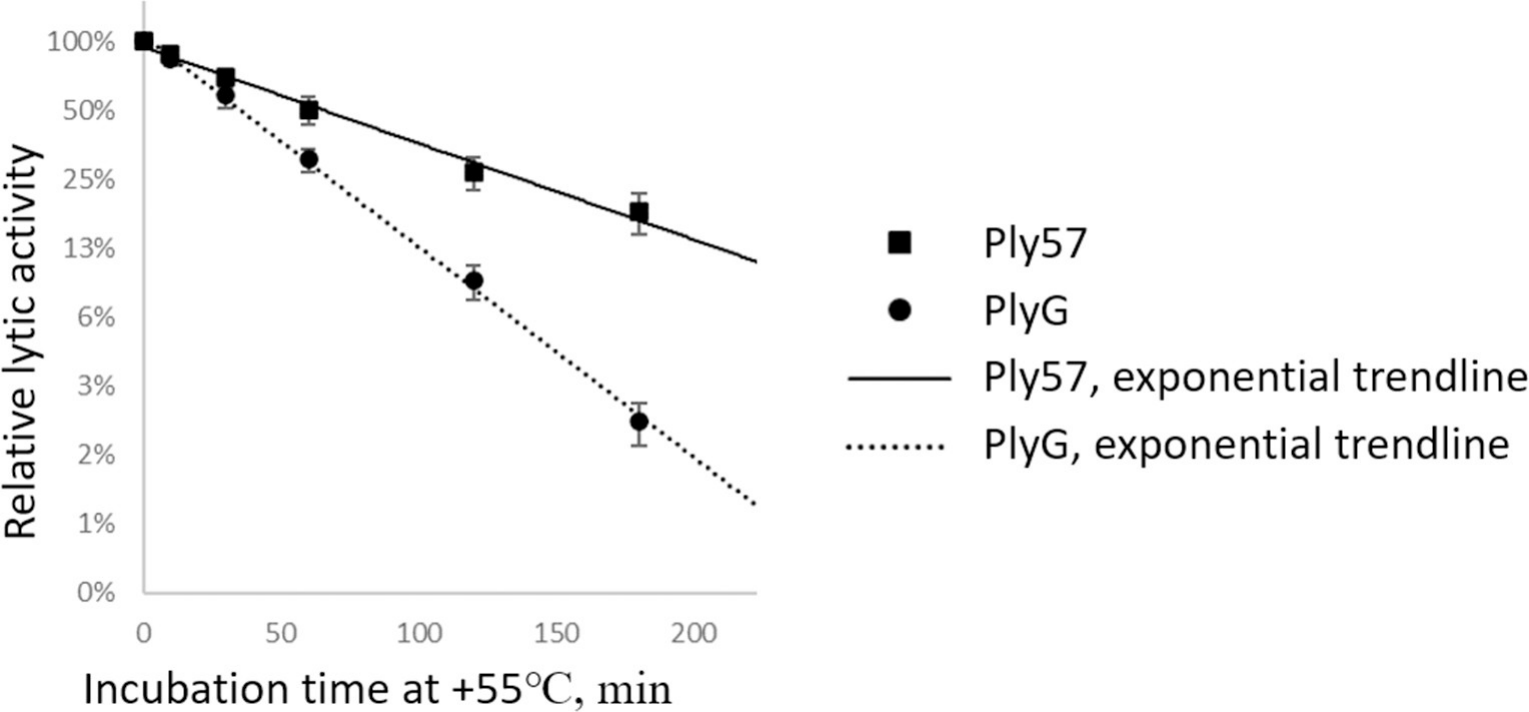
The thermal kinetic inactivation half-life experiment. Error bars indicate standard deviation values based on four replicates.

The N-terminal (aa residues 1–165) EAD of Ply57 shows a 53% identity to the EAD of the PlyG endolysin from phage Wβ, the best-characterized endolysin from the phages infecting the *B*. *cereus* group of bacteria [23]. Since the stability of bacteriolytic enzymes is one of the most essential criteria for the development of antimicrobial compounds, we have determined the relative thermostability of Ply57 compared to that of PlyG. For this purpose, the enzymes were incubated at 55°C and aliquots were removed at different time intervals. The residual bacteriolytic activity is exponentially dependent on the incubation time at 55°C, with the R^2^ values of 0.98 for Ply57 and 0.99 for PlyG (Fig 7). The thermostability assay has shown that Ply57 is two-fold more stable than PlyG under the experimental conditions. The time required for half-inactivation of the Ply57 bacteriolytic activity was 65±9 min whereas the half-inactivation time for PlyG was found to be 35±3 min.

## Discussion

The temperate *Bacillus* phage Izhevsk isolated and characterized in this work belongs to a distinct group of viruses within the *Siphoviridae* family along with the previously described phages Tsamsa [31] and PBC2 [22] and three other *Bacillus*-infecting viruses: Diildio, pW2 and vB_BcoS_136.

There have been many genome-based approaches proposed, aimed at establishing a universal scheme of phage taxonomical classification including those using concatenated phylogeny, shared protein content and pairwise genomic distances [25,47,48]. The nucleotide identity level of 95% is now used by the International Committee on Taxonomy of Viruses (ICTV) as a strict criterion for species demarcation, whereas the boundaries of higher taxa are less conservative.

In several extensive works, the phages of the order *Caudovirales* were assigned to one genus on the basis of shared protein content (>40%), similar morphology, whole-genome collinearity and shared key features [49–51]. Our comparative analyses have revealed that the group containing the four most closely related phages: Izhevsk, Tsamsa, Diildio and pW2, possesses 150 shared proteins (Table 2), i.e., 57.2% on average (in terms of the total number of proteins encoded in each of the genomes).

Although not all of these phages have been described in detail, their genomes have similar gene orders with highly identical DNA packaging, structural and replication genes (Fig 4) and possess the homologs of all six Izhevsk genes (ORFs 80, 208, 237, 238, 246, 247) predicted to have prophage-related functions, suggesting these are all temperate bacteriophages with the *Siphoviridae* morphology using short DTR packaging strategy. However, there are only 4 tRNA-coding genes in the genome of pW2, which differs significantly from the Izhevsk, Tsamsa and Diildio genomes harboring 18–19 tRNA genes.

The length and GC-content of the pW2 genome are two more characteristics deviating much stronger away from Izhevsk, Tsamsa and Diildio compared to more distantly related PBC2, which shares much less genes with the rest of the phages (Table 2). Thus, while considering the available set of genomes we could not decide whether pW2 and PBC2 can be placed into the same genus with Izhevsk, Tsamsa and Diildio; yet, the latter three phages can definitely be classified into a single genus in conformity with the accepted principles.

Among closely related phages, bacteriophage Izhevsk has the broadest specificity range: it is capable of infecting 97% of the tested *B*. *cereus sensu lato* strains (Table 1), whereas the specificity range of other related phages is quite narrow. The PBC2 phage was able to infect only one out of 11 tested *B*. *cereus sensu lato* strains [22]. Bacteriophage Tsamsa lysed 15 out of 38 (39%) tested *B*. *cereus sensu lato* strains, six of them being *B*. *anthracis* strains [31].

The Izhevsk endolysin Ply57 is active when produced recombinantly and shows a broad lytic spectrum. Ply57 belongs to the N-acetylmuramoyl-L-alanine amidase family and is highly similar to several other *Bacillus* phage endolysins, including the endolysins of phages Tsamsa (96.81% aa identity), pW2 (84.76%), PBC2 (80.88%) bg2 (79.94%) and Wβ (53% for EAD only). It has been reported that PlyG EAD contains a spore binding domain (SBD) allowing this molecule to degrade *B*. *anthracis* spores upon germination, even when CBD is completely deleted [52]. Structurally, SBD is a part of EAD but harbors amino acids involved in amidase catalytic activity (PDB 2L47). PlyG SBD is highly specific to *B*. *anthracis* spores [52]. Another characterized endolysin (from phage PBC2) also harbors a conservative SBD, which is specific to *B*. *cereus* spores, in contrast to the PlyG SBD [22]. We suppose that Ply57 can be active against germinating spores, similar to PBC2 endolysin, because the amino acid sequences of these endolysins corresponding to SBDs share 87% similarity (85% identity) (Fig 6).

All three endolysins of the Izhevsk, PBC2 and Tsamsa bacteriophages demonstrate a broad range of activity against the *B*. *cereus* group [22, 31]. PBC2 endolysin was active against *B*. *subtilis*, *B*. *megaterium*, *B*. *circulans*, *B*. *licheniformis*, *B*. *pumilus*, *L*. *monocytogenes*, *C*. *perfringens* and *S*. *aureus* [22]. Ply57 was also active against one of the two *B*. *subtilis* strains tested, as well as against *B*. *megaterium* and *B*. *flexus*, but not active against *B*. *pumilus* and *Enterococcus faecium* (Table 1). Ply57 and PBC2 endolysin may have different specificity due to the structural differences within the proximal SH3 domain and the linker connecting this domain to EAD. It has been recently shown that the length and flexibility of the linker part might affect thermal stability and bactericidal properties of the endolysin [53]. The linker part of Ply57, as well as that of bacteriophage Tsamsa endolysin, is six amino acids shorter than the linker in PBC2 endolysin.

Taken together, the broad specificity of phage Izhevsk, the high thermostability and broad specificity of its endolysin, Ply57, these objects are a useful platform for developing antibacterial agents and pathogen control tools to prevent bacterial contaminations caused by *B*. *cereus* group bacteria, including *B*. *anthracis*.

## Supporting information

S1 Fig Bacillus phage Izhevsk plaque morphology on 0.75% w/v LB overlay. (PDF)

S1 Raw images (PDF)

S1 Table Annotation of *Bacillus* phage Izhevsk. (PDF)

## References

[1] García P, Martínez B, Rodríguez L, Rodríguez A. Synergy between the phage endolysin LysH5 and nisin to kill *Staphylococcus aureus* in pasteurized milk. International journal of food microbiology. 2010 7 15;141(3):151–5. 10.1016/j.ijfoodmicro.2010.04.029 20537744

[2] Guo T, Xin Y, Zhang C, Ouyang X, Kong J. The potential of the endolysin Lysdb from *Lactobacillus delbrueckii* phage for combating *Staphylococcus aureus* during cheese manufacture from raw milk. Applied microbiology and biotechnology. 2016 4 1;100(8):3545–54. 10.1007/s00253-015-7185-x 26621799

[3] Gondil VS, Harjai K, Chhibber S. Endolysins as emerging alternative therapeutic agents to counter drug-resistant infections. International journal of antimicrobial agents. 2020 2 1;55(2):105844 10.1016/j.ijantimicag.2019.11.001 31715257

[4] Trigo G, Martins TG, Fraga AG, Longatto-Filho A, Castro AG, Azeredo J, et al Phage therapy is effective against infection by *Mycobacterium ulcerans* in a murine footpad model. PLoS neglected tropical diseases. 2013 4;7(4). 10.1371/journal.pntd.0002183 PMC3636042 23638204

[5] Nelson DC, Schmelcher M, Rodriguez-Rubio L, Klumpp J, Pritchard DG, Dong S, et al Endolysins as antimicrobials Advances in virus research 2012 1 1 (Vol. 83, pp. 299–365). Academic Press 10.1016/B978-0-12-394438-2.00007-4 22748813

[6] Loessner MJ. Bacteriophage endolysins—current state of research and applications. Current opinion in microbiology. 2005 8 1;8(4):480–7. 10.1016/j.mib.2005.06.002 15979390

[7] Carroll LM, Wiedmann M, Kovac J. Proposal of a taxonomic nomenclature for the *Bacillus cereus* group which reconciles genomic definitions of bacterial species with clinical and industrial phenotypes. Mbio. 2020 2 25;11(1). 10.1128/mBio.00034-20 32098810 PMC7042689

[8] Kumari S, Sarkar PK. Prevalence and characterization of *Bacillus cereus* group from various marketed dairy products in India. Dairy science & technology. 2014 9 1;94(5):483–97.

[9] Gundogan N, Avci E. Occurrence and antibiotic resistance of *Escherichia coli*, *Staphylococcus aureus* and *Bacillus cereus* in raw milk and dairy products in Turkey. International journal of dairy technology. 2014 11;67(4):562–9.

[10] Owusu-Kwarteng J, Wuni A, Akabanda F, Tano-Debrah K, Jespersen L. Prevalence, virulence factor genes and antibiotic resistance of *Bacillus cereus sensu lato* isolated from dairy farms and traditional dairy products. BMC microbiology. 2017 12;17(1):65 10.1186/s12866-017-0975-9 28288581 PMC5348786

[11] Bardell D. An 1898 report by Gamaleya for a lytic agent specific for *Bacillus anthracis*. Journal of the history of medicine and allied sciences. 1982 4 1;37(2):222–5. 10.1093/jhmas/xxxvii.2.222 6806352

[12] Gillis A, Mahillon J. Phages preying on *Bacillus anthracis*, *Bacillus cereus*, and *Bacillus thuringiensis*: past, present and future. Viruses. 2014 7;6(7):2623–72. 10.3390/v6072623 25010767 PMC4113786

[13] Piligrimova EG, Kazantseva OA, Nikulin NA, Shadrin AM. *Bacillus* Phage vB_BtS_B83 Previously Designated as a Plasmid May Represent a New *Siphoviridae* Genus. Viruses. 2019 7;11(7):624 10.3390/v11070624 31284652 PMC6669507

[14] Sambrook J, Fritsch EF, Maniatis T. Molecular cloning: a laboratory manual. Cold spring harbor laboratory press; 1989.

[15] Bankevich A, Nurk S, Antipov D, Gurevich AA, Dvorkin M, Kulikov AS, et al SPAdes: a new genome assembly algorithm and its applications to single-cell sequencing. Journal of computational biology. 2012 5 1;19(5):455–77. 10.1089/cmb.2012.0021 22506599 PMC3342519

[16] Brettin T, Davis JJ, Disz T, Edwards RA, Gerdes S, Olsen GJ, et al RASTtk: a modular and extensible implementation of the RAST algorithm for building custom annotation pipelines and annotating batches of genomes. Scientific reports. 2015 2 10;5:8365 10.1038/srep08365 25666585 PMC4322359

[17] Altschul SF, Gish W, Miller W, Myers EW, Lipman DJ. Basic local alignment search tool. Journal of molecular biology. 1990 10 5;215(3):403–10. 10.1016/S0022-2836(05)80360-2 2231712

[18] Altschul SF, Gish W, Miller W, Myers EW, Lipman DJ. BLAST: Basic local alignment search tool [Internet]. National Center for Biotechnology Information. U.S. National Library of Medicine; 1990 [cited 2020May22]. Available from: https://blast.ncbi.nlm.nih.gov/Blast.cgi 10.1016/S0022-2836(05)80360-2 2231712

[19] Zimmermann L, Stephens A, Nam SZ, Rau D, Kübler J, Lozajic M, et al A completely reimplemented MPI bioinformatics toolkit with a new HHpred server at its core. Journal of molecular biology. 2018 7 20;430(15):2237–43. 10.1016/j.jmb.2017.12.007 29258817

[20] Lowe TM, Eddy SR. tRNAscan-SE: a program for improved detection of transfer RNA genes in genomic sequence. Nucleic acids research. 1997 3 1;25(5):955–64. 10.1093/nar/25.5.955 9023104 PMC146525

[21] Stothard P, Wishart DS. Circular genome visualization and exploration using CGView. Bioinformatics. 2005 2 15;21(4):537–9. 10.1093/bioinformatics/bti054 15479716

[22] Kong M, Na H, Ha NC, Ryu S. LysPBC2, a novel endolysin harboring a Bacillus cereus spore binding domain. Appl. Environ. Microbiol. 2019 3 1;85(5):e02462–18. 10.1128/AEM.02462-18 30552194 PMC6384116

[23] Heselpoth RD, Owens JM, Nelson DC. Quantitative analysis of the thermal stability of the gamma phage endolysin PlyG: a biophysical and kinetic approach to assaying therapeutic potential. Virology. 2015 3 1;477:125–32. 10.1016/j.virol.2014.11.003 25432575

[24] Sullivan MJ, Petty NK, Beatson SA. Easyfig: a genome comparison visualizer. Bioinformatics. 2011 4 1;27(7):1009–10. 10.1093/bioinformatics/btr039 21278367 PMC3065679

[25] Meier-Kolthoff JP, Göker M. VICTOR: genome-based phylogeny and classification of prokaryotic viruses. Bioinformatics. 2017 11 1;33(21):3396–404. 10.1093/bioinformatics/btx440 29036289 PMC5860169

[26] Meier-Kolthoff JP, Auch AF, Klenk HP, Göker M. Genome sequence-based species delimitation with confidence intervals and improved distance functions. BMC bioinformatics. 2013 12 1;14(1):60 10.1186/1471-2105-14-60 23432962 PMC3665452

[27] Farris JS. Estimating phylogenetic trees from distance matrices. The American Naturalist. 1972 9 1;106(951):645–68.

[28] Rambaut A. A graphical viewer of phylogenetic trees 487 and a program for producing publication-ready figures [Internet]. FigTree. [cited 2020May22]. Available from: http://tree.bio.ed.ac.uk/software/figtree/

[29] Ackermann HW. Frequency of morphological phage descriptions in the year 2000. Archives of virology. 2001 5 1;146(5):843–57. 10.1007/s007050170120 11448025

[30] Russell DA. Sequencing, assembling, and finishing complete bacteriophage genomes In Bacteriophages 2018 (pp. 109–125). Humana Press, New York, NY. 10.1007/978-1-4939-7343-9_9 29134591

[31] Ganz HH, Law C, Schmuki M, Eichenseher F, Calendar R, Loessner MJ, et al Novel giant siphovirus from *Bacillus anthracis* features unusual genome characteristics. PLoS One. 2014;9(1). 10.1371/journal.pone.0085972 24475065 PMC3903500

[32] Blakely G, May G, McCulloch R, Arciszewska LK, Burke M, Lovett ST, et al Two related recombinases are required for site-specific recombination at *dif* and *cer* in E. coli K12. Cell. 1993 10 22;75(2):351–61. 10.1016/0092-8674(93)80076-q 8402918

[33] Huber KE, Waldor MK. Filamentous phage integration requires the host recombinases XerC and XerD. Nature. 2002 6;417(6889):656–9. 10.1038/nature00782 12050668

[34] Campos J, Martínez E, Izquierdo Y, Fando R. VEJ*φ*. A novel filamentous phage of *Vibrio cholerae* able to transduce the cholera toxin genes. Microbiology. 2010 1 1;156(1):108–15. 10.1099/mic.0.032235-0 19833774

[35] Midonet C, Das B, Paly E, Barre FX. XerD-mediated FtsK-independent integration of TLC*ϕ* into the *Vibrio cholerae* genome. Proceedings of the National Academy of Sciences. 2014 11 25;111(47):16848–53. 10.1073/pnas.1404047111 25385643 PMC4250166

[36] Yuan Y, Peng Q, Wu D, Kou Z, Wu Y, Liu P, et al Effects of actin-like proteins encoded by two *Bacillus pumilus* phages on unstable lysogeny, revealed by genomic analysis. Appl. Environ. Microbiol. 2015 1 1;81(1):339–50. 10.1128/AEM.02889-14 25344242 PMC4272706

[37] Sakaguchi Y, Hayashi T, Kurokawa K, Nakayama K, Oshima K, Fujinaga Y, et al The genome sequence of *Clostridium botulinum* type C neurotoxin-converting phage and the molecular mechanisms of unstable lysogeny. Proceedings of the National Academy of Sciences. 2005 11 29;102(48):17472–7. 10.1073/pnas.0505503102 16287978 PMC1283531

[38] Edgell DR. Free-standing homing endonucleases of T-even phage: freeloaders or functionaries? Homing endonucleases and Inteins 2005 (pp. 147–160). Springer, Berlin, Heidelberg.

[39] Chevalier BS, Stoddard BL. Homing endonucleases: structural and functional insight into the catalysts of intron/intein mobility. Nucleic acids research. 2001 9 15;29(18):3757–74. 10.1093/nar/29.18.3757 11557808 PMC55915

[40] Bell-Pedersen D, Quirk S, Clyman J, Belfort M. Intron mobility in phage T4 is dependent upon a distinctive class of endonucleases and independent of DNA sequences encoding the intron core: mechanistic and evolutionary implications. Nucleic Acids Research. 1990 7 11;18(13):3763–70. 10.1093/nar/18.13.3763 2165250 PMC331075

[41] Landthaler M, Lau NC, Shub DA. Group I intron homing in *Bacillus* phages SPO1 and SP82: a gene conversion event initiated by a nicking homing endonuclease. Journal of bacteriology. 2004 7 1;186(13):4307–14. 10.1128/JB.186.13.4307-4314.2004 15205433 PMC421625

[42] Tock MR, Dryden DT. The biology of restriction and anti-restriction. Current opinion in microbiology. 2005 8 1;8(4):466–72. 10.1016/j.mib.2005.06.003 15979932

[43] Reeve JN, Shaw JE. Lambda encodes an outer membrane protein: the *lom* gene. Molecular and General Genetics MGG. 1979 1 1;172(3):243–8. 10.1007/BF00271723 45607

[44] Sun X, Göhler A, Heller KJ, Neve H. The *ltp* gene of temperate *Streptococcus thermophilus* phage TP-J34 confers superinfection exclusion to *Streptococcus thermophilus* and *Lactococcus lactis*. Virology. 2006 6 20;350(1):146–57. 10.1016/j.virol.2006.03.001 16643978

[45] Erez Z, Steinberger-Levy I, Shamir M, Doron S, Stokar-Avihail A, Peleg Y, et al Communication between viruses guides lysis–lysogeny decisions. Nature. 2017 1;541(7638):488–93. 10.1038/nature21049 28099413 PMC5378303

[46] Humann J, Lenz LL. Bacterial peptidoglycan-degrading enzymes and their impact on host muropeptide detection. Journal of innate immunity. 2009;1(2):88–97. 10.1159/000181181 19319201 PMC2659621

[47] Low SJ, Džunková M, Chaumeil PA, Parks DH, Hugenholtz P. Evaluation of a concatenated protein phylogeny for classification of tailed double-stranded DNA viruses belonging to the order Caudovirales. Nature microbiology. 2019 8;4(8):1306–15. 10.1038/s41564-019-0448-z 31110365

[48] Rohwer F, Edwards R. The Phage Proteomic Tree: a genome-based taxonomy for phage. Journal of bacteriology. 2002 8 15;184(16):4529–35. 10.1128/jb.184.16.4529-4535.2002 12142423 PMC135240

[49] Adriaenssens EM, Edwards R, Nash JH, Mahadevan P, Seto D, Ackermann HW, et al Integration of genomic and proteomic analyses in the classification of the *Siphoviridae* family. Virology. 2015 3 1;477:144–54. 10.1016/j.virol.2014.10.016 25466308

[50] Lavigne R, Darius P, Summer EJ, Seto D, Mahadevan P, Nilsson AS, et al Classification of *Myoviridae* bacteriophages using protein sequence similarity. BMC microbiology. 2009 12 1;9(1):224 10.1186/1471-2180-9-224 19857251 PMC2771037

[51] Lavigne R, Seto D, Mahadevan P, Ackermann HW, Kropinski AM. Unifying classical and molecular taxonomic classification: analysis of the *Podoviridae* using BLASTP-based tools. Research in microbiology. 2008 6 1;159(5):406–14. 10.1016/j.resmic.2008.03.005 18555669

[52] Yang H, Wang DB, Dong Q, Zhang Z, Cui Z, Deng J, et al Existence of separate domains in lysin PlyG for recognizing *Bacillus anthracis* spores and vegetative cells. Antimicrobial Agents and Chemotherapy. 2012 10 1;56(10):5031–9. 10.1128/AAC.00891-12 22802245 PMC3457386

[53] Yang H, Luo D, Etobayeva I, Li X, Gong Y, Wang S, et al Linker editing of pneumococcal lysin ClyJ conveys improved bactericidal activity. Antimicrobial Agents and Chemotherapy. 2020 1 27;64(2). 10.1128/AAC.01610-19 31767724 PMC6985707

