# Supplementary figures and images for: Bacillus-infecting bacteriophage Izhevsk harbors thermostable endolysin with broad range specificity
Source: PLoS One. 2020 Nov 24;15(11):e0242657. doi: 10.1371/journal.pone.0242657 (PMC7685451; doi:10.1371/journal.pone.0242657)

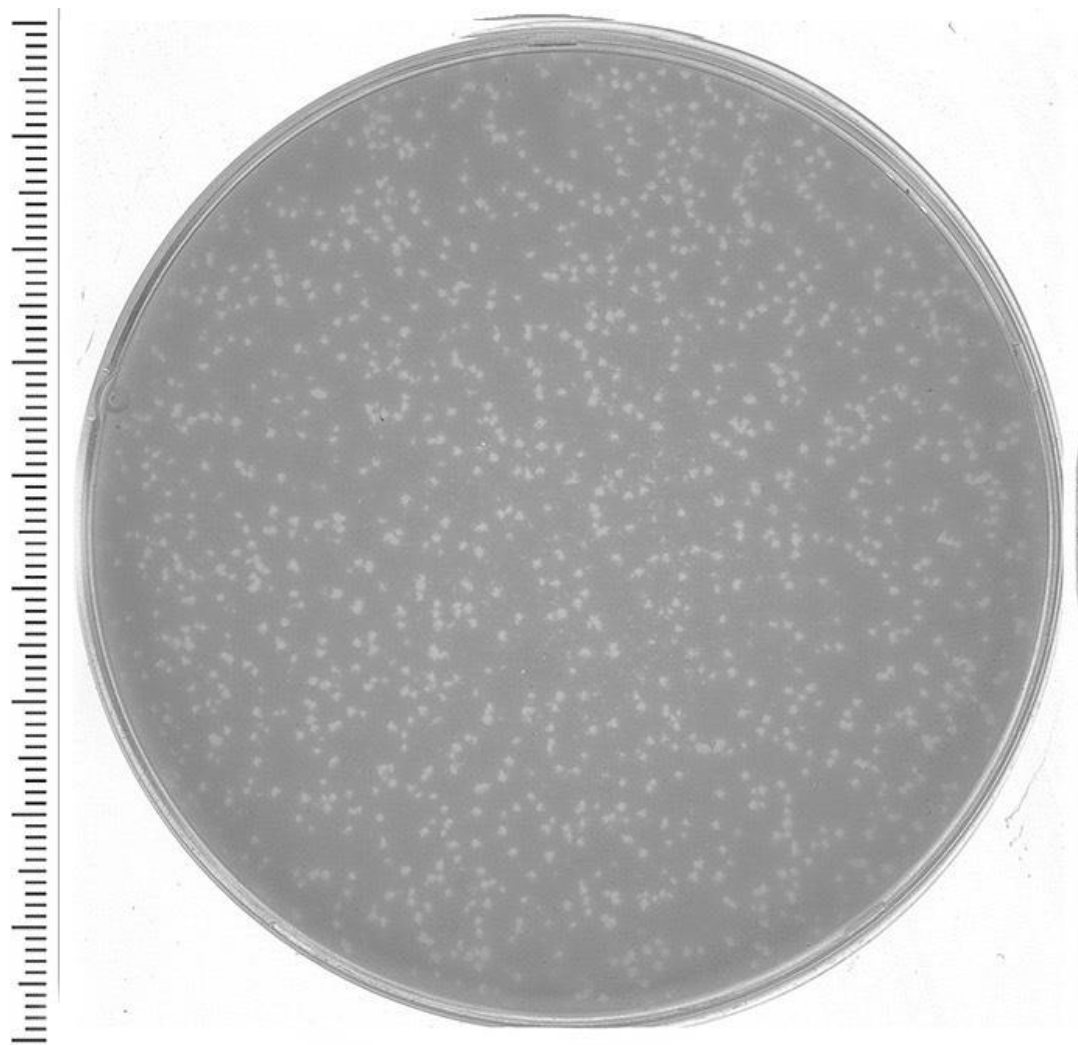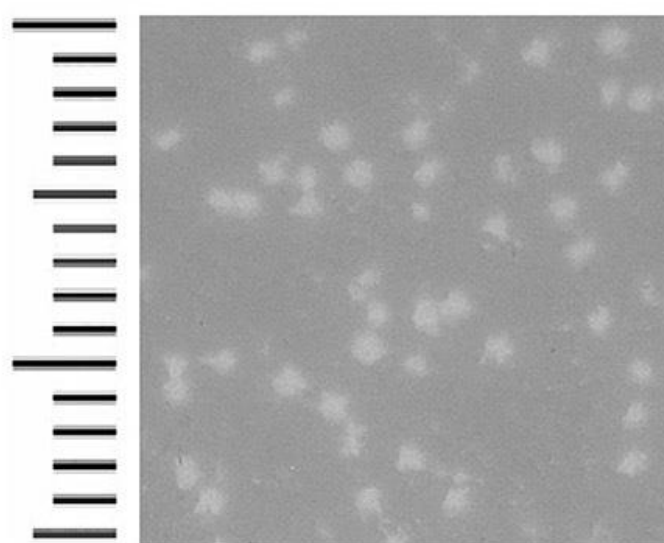

S1 Fig. *Bacillus* phage Izhevsk plaque morphology on 0.75% w/v LB overlay.

Supplement: S1 Fig — (PDF) [file pone.0242657.s001.pdf]
