# Supplementary material for: Bacillus-infecting bacteriophage Izhevsk harbors thermostable endolysin with broad range specificity
Source: PLoS One. 2020 Nov 24;15(11):e0242657. doi: 10.1371/journal.pone.0242657 (PMC7685451; doi:10.1371/journal.pone.0242657)
Supplement: S1 Raw images — (PDF) [file pone.0242657.s002.pdf]

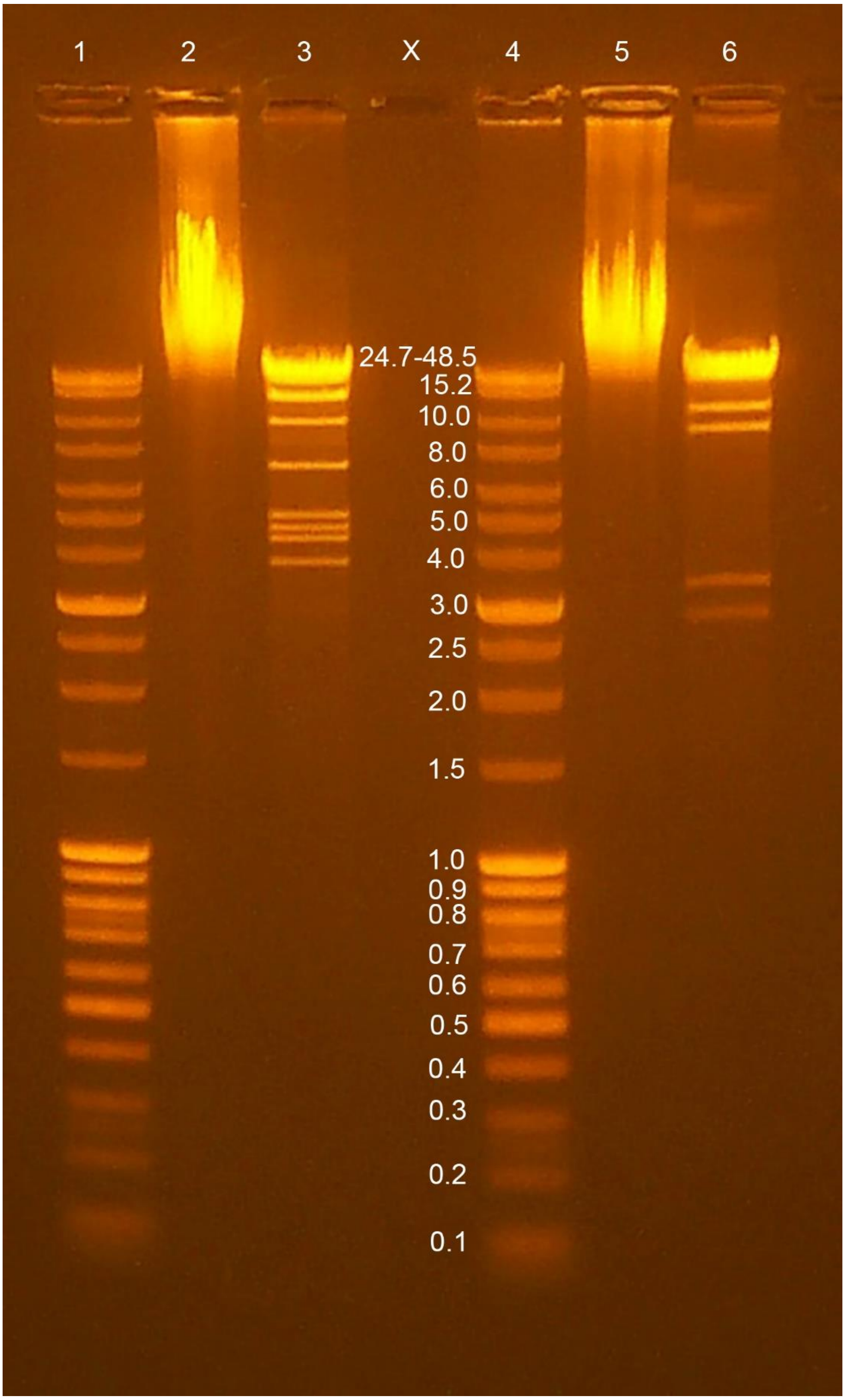

Phage DNA digested with restriction endonucleases PacI (lane 3) and AfeI (lane 6). Lanes 1, 4 – molecular weight markers, 2, 5 – intact phage DNA. This is the original gel image used to generate Fig.2 in the main text. Kodak EDAS 290 Gel Documentation System (“Kodak”) was used to capture the image.
