# Supplementary material for: Bacillus-infecting bacteriophage Izhevsk harbors thermostable endolysin with broad range specificity
Source: PLoS One. 2020 Nov 24;15(11):e0242657. doi: 10.1371/journal.pone.0242657 (PMC7685451; doi:10.1371/journal.pone.0242657)
Supplement: S1 Table — (PDF) [file pone.0242657.s003.pdf]

Table S1. Annotation of *Bacillus* phage Izhevsk

ORF№	Strand	Start\stop codons	Blast results		Conserved domains		HHpred	Predicted function
			Name	E-val	Name	E-val		
1	-	3109/1769	hp*				DnaJ-class molecular chaperone with C-terminal Zn finger domain 98.97/7.7e^{-12} Cysteine-rich domain of the chaperone protein DnaJ 98.17/2.7e^{-8}	Putative DnaJ domain-containing protein
2	-	4073/3138	hp		PRK10819 (118-216)	2.8e^{-5}		hp (a/a sequence is identical to 63)
					predic_Ig_block (126-242)	7.89e^{-5}		
3	-	4318/4160	hp					hp
4	-	4429/4337	hp					hp
5	-	4839/4444	hp		PRK10819 (118-216)	2.8e^{-5}		hp (a/a sequence is identical to 60)
					predic_Ig_block (126-242)	7.89e^{-5}		
6	-	5174/4932	hp					hp
7	-	5306/5190	hp					hp
8	-	5599/5303	hp					hp
9	+	6102/6227	hp					hp
10	+	7346/7675	hp					hp
11	+	7705/8061	hp					hp
12	+	8437/8643	hp					hp
13	+	8706/9374	Serine-threonine kinase [*Bacillus* virus Finn]	2e^{-5}	SPS1 (33-158)	3.56e^{-7}		Serine/threonine protein kinase
14	+	9583/9978	hp				Peptidase_C23; Carlavirus endopeptidase 96.5/0.00079 Peptidase_C34;	Putative peptidase

							Putative closterovirus papain-like endopeptidase 95.79/0.0066 bacteriocin_ABC; ABC-type bacteriocin transporter. 87.28/13	
15	+	10055/10438	Putative membrane lipoprotein [*Paenibacillus* phage Yerffej]	$6e^{-28}$	DUF3862 (51-126)	$3.25e^{-8}$	BamE; Outer membrane protein assembly factor BamE, lipoprotein component of the BamABCDE complex 99.03/$6.4e^{-11}$	Predicted signal peptide-containing lipoprotein (Cleavage site between pos. 21 and 22: AVA-CT. Probability: 0.9990)
16	+	10584/10763	Anti-restriction-like protein [*Bacillus* phage Shbh1]	$7e^{-31}$				hp
17	+	10853/11353	Anti-restriction-like protein [*Bacillus* phage Shbh1]	$7e^{-31}$			ocr; DNA mimic ocr 99.62/$2.8e^{-18}$ a.159.3.1 (A:) B-form DNA mimic Ocr {Bacteriophage T7 [TaxId: 10760]} 99.59/$6.5e^{-18}$	Putative T7 Ocr-like protein, type I DNA restriction enzymes inhibitor
18	+	11458/11853	hp					hp
19	+	11866/12312	hp					hp
20	+	12380/12724	hp					hp
21	+	12771/12923	hp					Predicted signal peptide-containing lipoprotein (Cleavage site between pos. 14 and 15: MQG-CW. Probability: 0.6204)
22	+	13053/14078	M20/M25/M40 family metallo-hydrolase [*Bacillus* phage pW2]	$7e^{-165}$	FrvX (1-244)	$5.37e^{-19}$		Peptidase, Putative metallo-aminopeptidase
					M42_Frv (8-244)	$4.67e^{-14}$		
					PRK06133 (8-145)	$1.72e^{-08}$		
					Peptidase_M42 (103-243)	$5.97e^{-08}$		

23	+	14170/14508	hp					hp
24	+	14520/14810	hp					hp
25	+	14875/15279	hp					hp
26	+	15281/15745	hp					hp
27	+	15808/15909	hp					hp
28	+	16028/16345	hp					hp
29	+	16402/16692	hp					hp
30	+	16689/16925	hp					hp
31	+	16922/17065	hp					hp
32	+	17070/17306	hp					hp
33	+	17311/17478	hp					hp
34	+	17507/17689	hp					hp
35	+	17694/17789	hp					predicted signal peptide-containing lipoprotein (Cleavage site between pos. 16 and 17: AFS-CI. Probability: 0.7039)
36	+	17853/18227	hp					hp
37	+	18300/18650	hp					hp
38	+	18694/18864	hp					hp
39	+	18866/19054	hp					hp
40	+	19059/19238	hp					hp
41	+	19267/19386	hp					hp
42	+	19414/19590	hp					hp
43	+	19602/19781	hp					hp
44	+	19812/19979	hp					hp
45	+	20061/20489	hp					hp
46	+	20498/20677	hp					hp
47	+	20691/20969	hp					hp
48	+	20981/21193	hp					hp
49	+	21210/21611	hp					hp
50	+	21641/21835	hp					hp
51	+	21867/21956	hp					hp
52	+	21972/22145	hp					hp
53	+	22287/22502	hp					hp

54	+	22562/22984	hp					hp
55	+	22981/23190	hp					hp
56	+	23210/23431	hp					hp
57	+	23593/23790	hp					hp
58	+	23771/23941	hp					hp
59	+	24048/24734	Nicotinamide mononucleotide transporter [*Bacillus* phage PBC2]	$4e^{-126}$	NMN_transporter (33-220)	$1.13e^{-36}$	Ribosyl nicotinamide transporter, PnuC-like OS=Bacillus sp. 100/$2.9e^{-68}$	PnuC-like nicotinamide mononucleotide transporter
60	+	24736/24864						hp
61	+	24877/25572	AP2 domain-containing protein [*Desulfobacteraceae bacterium*]	$1e^{-37}$			Intron-encoded homing endonuclease0 I-HmuI {Bacteriophage} SPO1 93.48/0.095 HNH_3 ; HNH endonuclease 92.45/0.12	Putative homing endonuclease
62	+	25565/26593	Multifunctional transcriptional regulator/nicotinamide-nucleotide adenylyltransferase/ribosylnicotinamide kinase NadR [*Bacillus* phage pW2]	0.0	PRK08099 (1-341)	$2.17e^{-72}$		Nicotinamide-nucleotide adenylyltransferase, NadR type
					nadR_NMN_Atrans (3-330)	$6.67e^{-68}$		
					AAA_28 (170-328)	$3.64e^{-43}$		
					NadR3 (163-341)	$8.82e^{-41}$		
					NMNAT_NadR (5-164)	$4.22e^{-20}$		
63	+	26746/27054	hp					hp
64	+	27071/27427	hp					hp
65	+	27655/27831	hp		DUF2536 (8-51)	$7.13e^{-3}$		hp
66	+	27845/28255	Putative lipoprotein [*Bacillus* phage pW2]	$3e^{-89}$				Predicted signal peptide-containing lipoprotein (Cleavage site between pos. 23 and 24: LAG-CG. Probability: 0.9960)

67	+	28260/28457	hp					hp
68	+	28486/28677	hp					hp
69	+	28708/28956	hp					hp
70	+	28956/29171	hp					hp
71	+	29225/29491	hp					hp
72	+	29488/29793	hp					hp
73	+	29790/30020	hp					hp
74	+	30060/30473	hp				SH3_15 ; Mind bomb SH3 repeat domain 98.27/2.6e-8 IDEAL ; IDEAL domain 98.26/1.6e^{-8} YpiB; Uncharacterized protein YpiB 98.11/7.4e^{-8}	hp
75	+	30531/31034	SprT-like family protein [*Bacillus* phage pW2]	2e^{-26}	PRK04351 (59-163)	5.79e^{-12}	SprT domain-containing protein OS *Bacillus mycoides* 100/2.8e^{-53}	SprT domain-containing protein
					SprT (31-157)	1.51e^{-8}		
76	+	31031/31357	hp					hp
77	+	31439/31876	hp					hp
78	+	31978/32241	Helix-turn-helix domain-containing protein [*Bacillus massiliogabonensis*]	5e^{-8}	XRE (39-87)	2.14e^{-3}	XRE family transcriptional regulator OS *Bacillus tequilensis* 99.91/1.1e^{-28}	HTH domain-containing XRE family protein
79	+	32314/32823	hp				Secreted protein OS *Rhodopirellula sp.* 91.36/0.3	Predicted signal peptide-containing lipoprotein (Cleavage site between pos. 20 and 21: LVG-CS. Probability: 0.9993)
80	+	33130/34143	DNA breaking-rejoining enzyme/site-specific recombinase [*Bacillus*	0.0	XerC (33-309)	4.8e^{-4}	XerC; Site-specific recombinase XerC 100/1.4e^{-30}	XerC tyrosine site-specific recombinase

			phage pW2]		INT_ICEBs1_C_like (153-217)	5.6e^{-4}	recomb_XerC; tyrosine recombinase XerC 100/1.6e^{-9} recomb_XerD; tyrosine recombinase XerD 100/1.9e^{-9}	
81	+	34161/34478	MULTISPECIES: helix-turn-helix domain-containing protein [*Bacillus cereus* group]	3e^{-7}			a.35.1.3 (A:) Antitoxin HigA {*Escherichia coli*}98.11/0.0000015 VapI; Plasmid maintenance system antidote protein VapI, contains XRE-type HTH domain 97.68/0.000043 Phage_CI_repr; Bacteriophage CI repressor 97.37/0.00011 lambda C1 repressor 97.2/0.00028	hp
82	+	34644/35024	hp					hp
83	+	35138/36319	Putative single-stranded DNA binding protein [*Bacillus* phage pW2]	3e^{-169}	SSB_OBF (138-181)	8.99e^{-4}		Single-stranded DNA-binding protein
84	+	36468/37640	p-loop containing nucleoside triphosphate hydrolase [*Bacillus* phage PBC2]	3e^{-162}	AAA_24 (21-245)<br>PRK03918 (264-352)	6.67e^{-21}<br>5.68e^{-3}	phage_P_loop; phage nucleotide-binding protein 99.49/2.2e^{-14}	Nucleotide-binding protein
85	+	37718/38479	Cell wall-binding protein [*Bacillus* phage	4e^{-126}	3D_containing_proteins (155-252)	1.11e^{-36}	3D domain protein 99.92/8.6e^{-30}	3D domain-containing protein

			pW2		Smc (27-130)	$4.23e^{-6}$		
					SMC_prok_A (28-148)	$7.16e^{-5}$		
					Tropomyosin_1 (43-113)	$8.71e^{-5}$		
86	+	38494/39027	Deoxynucleoside kinase [*Bacillus* phage PBC2]	$1e^{-76}$	dNK (3-155)	$1.24e^{-10}$		Deoxyribonucleoside kinase
					AAA_18 (4-123)	$3.43e^{-9}$		
					Tmk (1-131)	$1.33e^{-6}$		
87	+	39135/39728						hp
88	+	39747/41279	Replicative DNA helicase [*Bacillus* phage vB_BcoS-136]	0.0	DnaB (47-306)	$7.53e^{-8}$	Helicase OS *Brevibacillus laterosporus* 100/$6.4e^{-41}$	DnaB-type replicative DNA helicase
89	+	41332/42081	GIY-YIG nuclease family protein [*Bacillus thuringiensis*]	$8e^{-43}$	grpIintron_endo (6-242)	$2.93e^{-32}$	Group I intron endonuclease 100/$3.3e^{-35}$ Group I intron GIY-YIG endonuclease 99.85/$3.4e^{-25}$	Homing endonuclease
					GIY-YIG_HE_Tlr8p_PBC-V_like (4-97)	$1.34e^{-24}$		
					NUMOD3 (134-173)	$1.18e^{-7}$		
					GIYc (3-100)	$6.97e^{-6}$		
90	+	42091/43155	DNA primase [*Bacillus* phage pW2]	0.0	PRK08624 (6-345)	$2.62e^{-28}$	DNA primase OS *Bacteroidetes bacterium* 100/$1.1e^{-34}$	DnaG-type DNA primase
					DnaG (185-318)	$3.98e^{-11}$		
					TOPRIM_DnaG_primases (246-299)	$4.64e^{-6}$		
					Toprim_N (185-221)	$9.45e^{-5}$		
91	+	43270/43824						hp
92	+	43939/44181						hp
93	+	44600/46288	Single-stranded-DNA-specific exonuclease [*Bacillus* phage pW2]	0.0	recJ (22-558)	$7.03e^{-90}$		RecJ-type single-stranded DNA-specific exonuclease
					PRK11070 (9-455)	$1.88e^{-33}$		
					DHH (157-229)	$5.21e^{-4}$		
94	+	46300/46536						hp
95	+	46617/47363	GTP pyrophosphokinase [*Bacillus* phage pW2]	$2e^{-106}$	spoT_relA (38-225)	$9.72e^{-23}$	GTP pyrophosphokinase OS *Bacillus cereus* 100/$3e^{-77}$	SpoT-like bifunctional (p)ppGpp synthase/hydrolase
					PRK11092 (38-169)	$1.48e^{-18}$		
					SpoT (40-169)	$4.89e^{-17}$		
					HD_4 (38-169)	$3.25e^{-13}$		
96	+	47356/47523						hp
97	+	47588/47776						hp

98	+	47808/48020						hp
99	+	48025/48492	macro domain-containing protein [*Bacillus licheniformis*]	e^{-47}	Macro_Poa1p_like (1-141)	$2.62e^{-34}$	Appr-1-p processing protein OS *Bacillus pumilus* 100/$6.5e^{-43}$	Poa1p-like Appr-1”-p processing protein
					A1pp (1-137)	$1.79e^{-15}$		
			Appr-1-p processing protein [*Paenibacillus donghaensis*]	$2e^{-45}$	YmdB (1-152)	$4.40e^{-14}$		
					tk.4 (1-140)	$9.17e^{-13}$		
					Macro (19-137)	$6.27e^{-7}$		
100	+	48497/48796	hp					hp
101	+	48992/49345	hp				Putative small lipoprotein YifL OS *Oceanicaulis sp.* PT13A 92.54/0.13	Predicted signal peptide-containing lipoprotein (Cleavage site between pos. 22 and 23: LSG-CG. Probability: 0.9992)
102	+	49356/49766	hp					hp
103	+	49756/50313	GIY-YIG nuclease family protein [*Bacillus tropicus*]	$4e^{-21}$	GIY-YIG_UvrC_Cho (7-80)	$2.65e^{-14}$	Excinuclease ABC subunit C OS *Bacillus cereus* 99.43/$2.4e^{-16}$	Excinuclease
					UvrC (7-81)	$2.17e^{-8}$		
104	+	50310/50588	hp					Predicted signal peptide-containing lipoprotein (Cleavage site between pos. 19 and 20: LGA-CG. Probability: 0.9974)
105	+	50598/50753	hp					hp
106	+	50755/51099	hp					hp
107	+	51099/51338	hp					hp
108	+	51328/51606	hp		Spt20 (13-76)	$4.26e^{-3}$		hp
109	+	51636/51851	hp					hp
110	+	51865/51999	hp					hp
111	+	52004/52213	hp					hp
112	+	52332/52658	hp					hp
113	+	52714/52893	hp					hp
114	+	52906/53100	hp					hp
115	+	53105/53638	NUMOD4 motif family protein [*Clostridium botulinum*]	$7e^{-34}$	HNH_3 (63-107)	$3.45e^{-13}$	HNH homing endonuclease OS *Enterococcus* phage	HNH homing endonuclease
					NUMOD4 (2-54)	$1.57e^{-9}$		

							100/$7.6e^{-35}$	
116	+	53643/54095	hp					hp
117	+	54100/54843	NADAR family protein [Bacillus phage pW2]	$3e^{-128}$	NADAR (6-155)<br>DUF1768 (9-158)<br>ribofla_fusion (8-158)<br>ybiA (1-158)	$1.26e^{-50}$<br>$2.47e^{-48}$<br>$6.71e^{-47}$<br>$1.52e^{-34}$		NADAR superfamily protein
118	+	54836/55402	hp					hp
119	+	55407/55718	hp					hp
120	+	55745/55987	hp					hp
121	+	56001/56267	hp					hp
122	+	56261/56515	hp					hp
123	+	56589/57347	Putative nucleotidyltransferase [*Bacillus* phage vB_BcoS-136]	$3e^{-76}$	nrdC.11 (7-141)	8.64e-*4*	Nucleotidyl transferase OS *Mycobacterium* phage GenevaB15 95.68/0.0017	putative nucleotidyltransferase
124	+	57407/59362	DNA topoisomerase [*Bacillus* phage PBC2]	0.0	GyrB (2-649)<br>TOP2c (30-642)<br>TOPRIM_TopoIIA_GyrB (427-548)<br>DNA_gyraseB_C (578-639)	$6.75e^{-150}$<br>$1.52e^{-129}$<br>$3.68e^{-47}$<br>$7.06e^{-161}$		DNA gyrase subunit B
125	+	59408/61561	DNA gyrase subunit A [*Bacillus* phage vB_BcoS-136]	0.0	GyrA (4-714)<br>PRK05561 (4-688)<br>gyrA (4-713)<br>DNA_topoisoIV (28-449)<br>TOP4c (8-453)	$7.06e^{-161}$<br>$1.42e^{-152}$<br>$1.06e^{-140}$<br>$1.05e^{-108}$<br>$1.05e^{-108}$	DNA gyrase subunit A 100/$2.4e^{-97}$	DNA gyrase subunit A
126	+	61611/63089	Putative PcfJ-like protein [*Brevibacillus* phage Sundance]	$3e^{-71}$	PcfJ (336-478)	$2.48e^{-25}$	PcfJ-like protein 99.7/$8.3e^{-21}$	PcfJ-like protein
127	+	63160/63564	DUF1653 domain-containing protein [*Bacillus thuringiensis*]	$1e^{-7}$	DUF1653 (1-102)<br>COG4728 (3-109)	$1.44e^{-10}$<br>$2.61e^{-5}$		DUF1653 domain-containing protein

128	+	63744/64049	Putative ribonucleotide reductase [*Bacillus* phage PBC2]	$6e^{-2}$	nrdI (4-95)<br>Flavodoxin_NdrI (5-91)	$5.90e^{-31}$<br>$1.45e^{-27}$	NrdI protein 99.56/$2.8e^{-18}$	NrdI-like protein of class Ib ribonucleotide reductase operon
129	+	64046/64267	hp					hp
130	+	64283/66394	Ribonucleotide reductase [*Bacillus* phage PBC2]	0.0	RNR_1b_NrdE (6-703)<br>RNR_I (117-690)<br>Ribonuc_red_lgC (167-687)	0.0<br>0.0<br>0.0	Ribonucleoside-diphosphate reductase 100/$1.5e^{-221}$	NrdE-like class 1b ribonucleotide reductase, alpha subunit
131	+	66412/67467	Class 1b ribonucleoside-diphosphate reductase subunit beta [*Thermoactinomyces sp*. DSM 45892]	0.0	RNR_1b_NrdF (25-347)<br>Ribonuc_red_sm (28-308)<br>RNRR2 (28-316)	$1.01e^{-169}$<br>$6.04e^{-87}$<br>$9.21e^{-81}$	Ribonucleoside-diphosphate reductase subunit beta 100/$1e^{-101}$	NrdE-like class 1b ribonucleotide reductase, beta subunit
132	+	67550/67855	thioredoxin family protein [*Lentzea waywayandensis*]	$9e^{-6}$	TRX_family (9-77)<br>thioredoxin (11-62)	$8.21e^{-7}$<br>$3.07e^{-4}$	Thiol reductase thioredoxin 98.52/$3.5e^{-10}$	thioredoxin
133	+	67959/68891	dUTP diphosphatase [*Bacillus thuringiensis*]	$2e^{-57}$	dut (136-237)<br>Dut (137-241)<br>trimeric_dUTPase (156-242)	$6.80e^{-16}$<br>$4.64e^{-14}$<br>$3.17e^{-11}$	Deoxyuridine 5'-triphosphate nucleotidohydrolase 100/$1.3e^{-39}$	Trimeric dUTP diphosphatase
134	+	68990/69331	hp					hp
135	+	69383/69634	hp					hp
136	+	69648/70172	endonuclease [*Staphylococcus delphini*]	$1e^{-33}$	HNH_3 (63-108)<br>NUMOD4 (2-54)	$2.25e^{-10}$<br>$2.53e^{-6}$		HNH homing endonuclease
137	+	70184/70444	hp					Predicted signal peptide-containing lipoprotein (Cleavage site between pos. 21 and 22:

								MSG-CM. Probability: 0.9739)
138	+	70450/70611	hp					hp
139	+	70624/71175	Putative guanylate kinase [*Bacillus* phage pW2]	$4e^{-100}$	guanyl_kin (3-163)	$8.11e^{-39}$		Guanylate kinase
					GMPK (3-119)	$4.04e^{-32}$		
					GuKc (10-183)	$1.57e^{-25}$		
140	+	71188/71724	hp					hp
141	+	71728/71817	hp					hp
142	+	71830/72093	hp					hp
143	+	72098/72340	hp					Predicted signal peptide-containing lipoprotein (Cleavage site between pos. 17 and 18: LAG-CG. Probability: 0.9991)
144	+	72345/73070	MBL fold metallo-hydrolase [*Bacillus* phage pW2]	$2e^{-131}$	RNaseZ_MBL-fold (4-161)	$3.95e^{-18}$	Metal-dependent hydrolase OS *Bacillus* phage Bobb 100/$9.3e^{-80}$	MBL-fold metallohydrolase superfamily protein, putative RNaseZ-like tRNA maturase
					Lactamase_B (21-219)	$1.40e^{-10}$		
					RNase_Z (4-152)	$2.19e^{-9}$		
					PRK00055 (1-81)	$4.46e^{-9}$		
145	+	73072/73296	hp					hp
146	+	73296/73520	hp					hp
147	+	73521/73772	hp					hp
148	+	73927/74244	hp		PRK04326 (9-57)	$5.97e^{-3}$		hp
149	+	74266/74868	Nucleotidyltransferase domain-containing protein	$2e^{-26}$	NT_KNTase_like (3-48)	$4.14e^{-3}$	Putative nucleotidyltransferase 95.82/0.0023	Putative nucleotidyltransferase
150	+	74870/75136	Putative membrane lipoprotein [*Bacillus* phage vB_BmeM-Goe8]	$3e^{-31}$				Predicted signal peptide-containing lipoprotein (Cleavage site between pos. 19 and 20: LTG-CQ. Probability: 0.9984)
151	+	75143/75577	hp					hp
152	+	75589/75801	hp					hp
153	+	75785/76645	Putative subtilisin-like protein [*Bacillus* phage PBC2]	$2e^{-109}$	Peptidases_S8_Subtilisin_subset (42-266)	$4.10e^{-83}$		Serine peptidase S8 (subtilisin-like)
					Peptidase_S8 (40-265)	$5.40e^{-48}$		
					T7SS_mycosin (30-280)	$9.96e^{-47}$		
					AprE (24-284)	$1.49e^{-40}$		

154	+	76752/77912	hp		rnlA (64-197)	4.93e^{-15}	RNA ligase, T4 RnlA family. 100/2.5e^{-58}	RNA ligase (tRNA)
					RNA_lig_T4_1 (66-272)	4.37e^{-10}		
155	+	77909/78883	hp		AAA_33 (5-132)	8.87e^{-22}	bacter_Pnkp; polynucleotide kinase-phosphatase 98.62/1.3e^{-7}	AAA family ATPase domain-containing protein
					COG4639 (1-137)	1.58e^{-17}		
					pseT (1-127)	1.15e^{-12}		
					bacter_Pnkp (5-130)	9.29e^{-12}		
156	+	78896/79096	hp					hp
157	+	79128/79727	hp		ClpP (61-198)	6.25e^{-4}		Clp protease
158	+	79742/81046	hp		30 (11-422)	1.06e^{-47}	DNA_lig_bact; DNA ligase, ATP-dependent, PP_1105 family. 100/1.4e^{-31}	DNA ligase
					CDC9 (151-434)	3.61e^{-30}		
					Adenylation_kDNA_ligase_like (136-330)	2.91e^{-27}		
159	+	81058/81327	hp					hp
160		81328/81636	hp					hp
161	+	81636/82373	hp		grpIintron_endo (5-135)	9.41e^{-27}	group I intron endonuclease 99.7/1e^{-18}	Homing endonuclease
					GIY-YIG_HE_I-TevI_like (5-93)	2.02e^{-25}		
					GIYc (5-91)	2.61e^{-9}		
162	+	82374/83276	hp		RNA_ligase (27-124)	1.24e^{-6}	RNA ligase, Rnl2 family 100/1.5e-^{38}	RNA ligase
163		83336/83749	hp					hp
164	+	83763/84176	hp					hp
165	+	84181/84591	Hypothetical protein [*Bacillus* phage vB_BanS-Tsamsa]	9e^{-90}			Uncharacterized lipoprotein YehR, DUF1307 family [Function unknown]. 90.65/0.21 Lpp; Outer membrane murein-binding lipoprotein Lpp 85.53/5.2	Predicted signal peptide-containing lipoprotein Cleavage site between pos. 22 and 23: MVA-CA. Probability: 0.9956
			Hypothetical protein pW2_55 [*Bacillus* phage pW2]	6e^{-64}				
166	+	84593/84793	Hypothetical protein [*Bacillus* phage vB_BanS-Tsamsa]					Predicted signal peptide-containing lipoprotein signal peptide 20-21 LVA-CD. Probability: 0.9994
167	+	84837/85382	hp		DUF1273 (9-172)	2.4e^{-29}		DUF1273 domain-containing

					PRK13660 (35-143)	$1.49e^{-13}$		protein
168	+	85433/86290	hp		ribP_PPkin (9-261)<br>PrsA (9-257)<br>PRTases_typeI (137-256)	$2.74e^{-23}$<br>$1.29e^{-22}$<br>$3.29e^{-11}$		Phosphoribosylpyrophosphate synthetase (ribose-phosphate pyrophosphokinase)
169	+	86313/88031	Nicotinate phosphoribosyltransferase [*Bacillus thuringiensis*]	0.0	PRK09198 (10-571)<br>PBEF_like (10-517)	0.0<br>0.0	NAPRTase_put; putative nicotinate phosphoribosyltransferase. 100/$2.5e^{-50}$	Nicotinate phosphoribosyltransferase
170	+	88043/88204	hp					hp
171	+	88268/88705	hp		YqeY (4-144)	$2.59e^{-25}$		GatB/YqeY domain-containing protein
172	+	88791/89012	hp					hp
173	+	89028/89402	hp					Predicted signal peptide-containing lipoprotein Cleavage site between pos. 19 and 20: MVG-CG. Probability: 0.9992
174	+	89414/89806	hp				5'-nucleotidase 99.22/$2e^{-13}$ Polynucleotide kinase, phosphatase domain {Bacteriophage} T4 99.16/$2.9e^{-12}$	Putative 5'-nucleotidase
175	+	89841/90356	hp				PF01710.16 HTH_Tnp_IS630; Transposase 98.82/$6.1e^{-10}$ COG2826 Tra8; Transposase and inactivated derivatives, IS630 family [Mobilome: prophages, transposons]. 98.52/$9e^{-9}$	Putative IS630 family transposase (or inactivated derivative)
176	+	90384/90725	hp					hp
177	+	90774/91019	hp					hp

178	+	91040/91876	hp		Thymidylat_synt (7-276)	$7.99e^{-80}$	d.117.1.1 (A:) Thymidylate synthase {*Bacillus subtilis*} 100/$5.7e^{-60}$	Thymidylate synthase
179	+	91888/92127	hp					hp
180	+	92129/92656	hp		DHFR_1 (1-161)	$7.48e^{-65}$	c.71.1.1 (A:) Dihydrofolate reductase, prokaryotic type 99.94/$1.1e^{-28}$	Dihydrofolate reductase
181	+	92669/93316	hp		CLP_protease (2-179)	$3.77e^{-32}$	ATP-dependent Clp protease, proteolytic subunit 99.9/$2.6e^{-25}$	Clp protease
182	+	93407/93988	hp		YfbR (64-113).	$1.97e^{-5}$	5'-nucleotidase YfbR 99.44/$8e^{-15}$	Putative 5'-nucleotidase
183	+	93990/94655	hp		Tdk (7-190)	$3.02e^{-33}$	PF00265.18 TK ; Thymidine kinase 100/$1.8e^{-31}$	Thymidine kinase
184	+	94748/95317	hp					hp
185	+	95332/98442	hp		DnaE (1-936)	$1.98e^{-99}$	DnaE; DNA polymerase III, alpha subunit [Replication, recombination and repair]. 100/$1.6e^{-161}$	DNA polymerase III, alpha subunit
					PHP_PolIIIA_DnaE3 (7-212)	$4.56e^{-41}$		
186	+	98538/98744	hp		PncB_like (20-59)	$3.44e^{-3}$		hp
187	+	98818/99366	Holliday junction resolvase [*Bacillus* phage pW2]	5e-102	RuvC (8-160)	$3.57e^{-22}$	RuvC; Crossover junction endodeoxyribonuclease RuvC 99.73/$7.5e^{-19}$	RuvC Holliday-junction resolvase
188	+	99371/99865	hp					hp
189	-	100457/99954	hp					hp
190	-	101513/100560	hp		Laminin_G_3 Concanavalin A-like lectin/glucanases superfamily (50-207)	1.41e-7	b.29.1.15 (A:409-632) Trypanosoma sialidase, C-terminal domain 99.33/$5.9e^{-12}$ PF02973.16 Sialidase	concanavalin A-like lectin/glucanases superfamily protein, putative phage sialidase

							; Sialidase, N-terminal domain 98.71/3e^{-8}	
191	-	101810/101526	hp				Alpha-2-macroglobulin MG2 {Human (Homo sapiens)Ъ99.07/1.1e^{-10} YfaS; Uncharacterized conserved protein YfaS, alpha-2-macroglobulin family 98.42/7.4e^{-8}	hp
192	-	102271/101816	hp				Big_3_3; Bacterial Ig-like domain (group 3) 97.1/0.0026	Putative Ig-like domain-containing protein
193	-	102998/102324	hp				Big_3_3 ; Bacterial Ig-like domain (group 3) 97.18/0.00038	Putative Ig-like domain-containing protein
194	-	103191/103012	hp					hp
195	-	103547/103209	Putative holin [*Bacillus* phage PBC2]	2e^{-47}	Phage_holin_6_1 (8-103)	4.14e^{-10}		Holin
196	-	103940/103620	hp					hp
197	-	104951/104010	N-acetylmuramoyl-L-alanine amidase	6e^{-114}	CwlA (53-211)	3.48e^{-59}		N-acetylmuramoyl-L-alanine amidase
					Ami_2 (73-190)	4.48e^{-27}		
					PGRP (76-192)	5.72e^{-24}		
					Amidase_2 (71-192)	1.19e^{-22}		
198	-	105178/105026	hp					hp
199	-	105702/105247	hp					hp
200	-	108437/105705	Chaperone domain of endosialidase [*Bacillus* phage Kioshi]	0.0	Peptidase_S74 (808-858)	3.57e^{-6}		Endosialidase

201	-	112847/108462	Tail fiber protein [*Bacillus* phage pW2]	0.0			b.18.1.7 (A:) Xylan-binding domain {*Clostridium thermocellum* [TaxId: 1515]} 97.49/0.000067 b.18.1.14 (A:3-153) Cellulose-binding domain of cellulase C {*Cellulomonas fimi* [TaxId: 1708]} 97.54/0.000034	Putative receptor-binding protein
			Carbohydrate binding domain protein [*Exiguobacterium* phage vB_EalM-132]	$4e^{-67}$				
			receptor binding protein [*Bacillus* phage pW4]	$6e^{-19}$				
202	-	113566/112979	Putative endonuclease [*Bacillus* phage BCP78]	$2e^{-44}$	GIY-YIG_HE_Tlr8p_PBC-V_like (1-91)	$2.71e^{-27}$	grpIintron_endo; group I intron endonuclease. This model represents one subfamily of endonucleases containing the endo/excinuclease amino terminal domain, pfam01541 at its amino end. $99.76/1e^{-17}$	Homing endonuclease
			putative SegB homing endonuclease [*Staphylococcus* virus PH15]	$5e^{-19}$	grpIintron_endo (3-146)	1.82e-22		
			GIY-YIG catalytic domain-containing endonuclease [*Paramecium bursaria Chlorella* virus Can18-4	$2e^{-18}$	HTH_Hin_like (148-182)	$8.97e^{-5}$		
203	-	114667/113567	Putative tail fiber [*Bacillus* phage phiNIT1	$4e^{-58}$	34 (46-197)	$1.89e^{-3}$	Membrane glycoprotein LIG-1 [Signal transduction mechanisms] 95.8/ 0.37 Predicted transmembrane protein of the immunoglobulin family of cell adhesion molecules [General function prediction only] 92.91/1.8	Putative long tail fiber protein

204	-	117350/114687	Putative tail fiber protein [*Bacillus* phage PBC4]	$2e^{-27}$	Prophage_tail (44-251, 399-500)	$9.64e^{-6}$	PF06605.11 ; Prophage_tail ; Prophage endopeptidase tail 99.78/7.2e-20 COG4379; Mu-like prophage tail protein gpP [Mobilome: prophages, transposons]. 98.2/0.0000087	Putative tail endopeptidase
						$1.44e^{-5}$		
					Smc (272-4461)	$4.96e^{-5}$		
205	-	117852/117367	hp		Peptidase_C92 (3-148)	$4.50e^{-9}$	Peptidase_C92 ; Permuted papain-like amidase enzyme, YaeF/YiiX, C92 family 99.8/ $4.4e^{-23}$ RAT; Lecithin retinol acyltransferase 97.54/0.000017	Permuted papain-like amidase
206	-	118700/117930	Select seq gb|AZU98924.1| tail protein [*Bacillus* phage pW2]	$5e^{-139}$	YomH (10-250)	$4.04e^{-13}$	Sipho_tail ; Phage tail protein 99.91 $2.3e^{-25}$ Baseplate; Baseplate protein 91.81/3.3 Tail_tube ; Phage tail tube protein 83.96/13	Tail protein
					Sipho_tail (83-254)	$8.35e^{-3}$		
207	-	128089/118739	Putative tail tape measure protein [*Bacillus* phage pW2]	0.0	tape_meas_TP901 (645-992)	$4.49e^{-36}$	tape_meas_TP901; phage tail tape measure protein, 98.39/$6.4e^{-7}$	Tape measure protein
					PhageMin_Tail (685-887)	$2.07e^{-20}$		
					SMC_prok_A (2529-2842)	$1.38e^{-10}$		
208	-	129147/128167	Putative site specific recombinase [*Bacillus* phage pW2]	0.0	recomb_XerC (28-311)	$3.74e^{-18}$	XerC; Site-specific recombinase XerC [Replication,	Xer family site-specific tyrosine recombinase, putative XerC recombinase
					DNA_BRE_C (156-312)	$2.71e^{-19}$		

					XerD (22-311)	$8.69e^{-15}$	recombination and repair] $100/9.7e^{-32}$ recomb_XerD; tyrosine recombinase XerD. $100/2e^{-30}$	
209	-	129657/129157	Putative structural protein [*Bacillus* phage PBC2]	$4e^{-40}$			COG5005; Mu-like prophage protein gpG HK97-gp10_like ; Bacteriophage HK97-gp10, putative tail-component 50.37/85	Tail completion protein
210	-	130153/129638	hp					hp
211	-	131080/130337	hp				maj_tail_phi13; phage major tail protein, phi13 family. 74.9/21 Phage_TTP_1 ; Phage tail tube protein 63.49/110	Tail tube protein (major tail protein)
212	-	131979/131131	hp				Phage_TAC_7 ; Phage tail assembly chaperone proteins 21.75/150	Putative tail assembly chaperone
213	-	132473/131979	hp					hp
214	-	133243/132473	hp				gp16_SPP1; phage head-tail adaptor, putative, SPP1 family 90.09/2 COG5614; Bacteriophage head-tail adaptor [Mobilome: prophages, transposons]	Head completion protein

							89.84/2	
215	-	133566/133246	hp					hp
216	-	134027/133563	hp					hp
217	-	134753/134043	hp					hp
218	-	135832/134837	hp				d.183.1.1 (A:104-383) Major capsid protein gp5 {Bacteriophage HK97 [TaxId: 37554]} 97.78/0.000012 Phage_capsid; Phage capsid family 97.53/0.000023 capsid_maj_N4; major capsid protein, N4-gp56 family. 97.2/0.0046	Major capsid protein
219	-	136388/135864	hp					hp
220	-	137820/136417	hp				Peptidase_S78; Caudovirus prohead serine protease 91.47/7.4 Phage_GPO ; Phage capsid scaffolding protein (GPO) serine peptidase 90.06/2.8	Putative prohead protease
221	-	139356/137848	hp				cl19194: Phage_portal Superfamily. 99.91/$1.8e^{-26}$ portal_lambda; phage portal protein, lambda family.	Portal protein

	-						$99.8/7.4e^{-21}$ Phage_portal_2 ; Phage portal protein, lambda family $99.72/3e^{-20}$	
222	-	141065/139371	Putative terminase ATPase subunit [*Bacillus* phage pW2]	0.0			Terminase_1; Phage Terminase $100/9.8e^{-32}$ COG5323; Large terminase phage packaging protein [Mobilome: prophages, transposons]. $100/1.5e^{-30}$	Large terminase subunit
			Putative terminase ATPase subunit [*Bacillus* phage PBC2]	0.0				
223	-	141942/141043	hp				sm_term_P27; phage terminase, small subunit, putative, P27 family. This model describes a distinct family of phage (and integrated prophage) putative terminase small subunit. 70.59/54 PF05119.12 Terminase_4; Phage terminase, small subunit 46/170	Putative small terminase
224	-	142659/142099	hp				COG2842; Bacteriophage DNA transposition protein, AAA+ family ATPase $98.29/9.3e^{-8}$ c.37.1.1 (A:1-125,A:161-217) Adenylate kinase	hp

							{*Bacillus globisporus* [TaxId: 1459]} 98.09/5.5e-7 c.37.1.20 (A:17-254) Holliday junction helicase RuvB {*Thermotoga maritima* [TaxId: 2336]} 97.78/0.0000061	
225	-	143924/142683	Putative metallophophatase [*Bacillus* phage PBC2]	0.0	MPP_YfcE (313-377)	$4e^{-6}$		metallophosphatase
226	-	144147/143944					d.368.1.1 (A:2-63) Uncharacterized protein YonK {*Bacillus subtilis* [TaxId: 1423]} 99.9/$5.4e^{-27}$	hp
227	-	144473/144255						hp
228	-	145957/144515	gp165 [*Listeria* virus P100]	$2e^{-96}$	DUF2828 (14-468)	$3.8e^{-80}$	DUF2828; Domain of unknown function (DUF2828) 100/$5.9e^{-79}$	DUF2828 domail-containing protein
					VWA (291-387)	$9.22e^{-6}$		
					COG2425 (36-391)	$4.26e^{-3}$		
229	-	148026/147487	Select seq gb|ALP46693.1| HNH_3 endonuclease [*Bacillus* phage BM5]	$1e^{-18}$			d.4.1.3 (M:1-105) Intron-encoded homing endonuclease I-HmuI {Bacteriophage SPO1 [TaxId: 10685]} 99.05/$2.5e^{-12}$ InsA; Transposase [Mobilome: prophages, transposons]. 98.07/	Homing endonuclease

							$7.6e^{-8}$	
230	-	150620/149385	Phosphate starvation-inducible protein [*Bacillus* phage pW2]	0.0	YlaK (3-411)	$1.86e^{-31}$	YlaK; Predicted ribonuclease YlaK, contains NYN-type RNase and PhoH-family ATPase domains. $100/1.3e^{-37}$	PhoH2 protein, RNA helicase-ribonuclease
					PhoH (209-411)	$1.69e^{-19}$		
					PINc (1-112)	$1.10e^{-4}$		
231	-	150946/150641			BHL (4-98)	$6.37e^{-9}$	a.55.1.1 (A:) Integration host factor alpha subunit (IHFA) {*Escherichia coli* [TaxId: 562]} $99.83/7.5e^{-22}$	DNA-binding protein
					HimA (4-101) (HU_IHF Superfamily)	$1.33e^{-8}$		
					Bac_DNA_binding (5-97)	$2.48e^{-8}$		
232	-	151247/150951	hp					hp
233	-	151603/151247	hp					hp
234	-	152088/151660	hp				MalT; ATP-, maltotriose- and DNA-dependent transcriptional regulator MalT $98.07/1.6e^{-7}$ reg_near_HchA; LuxR transcriptional regulator family. $97.99/4e^{-7}$	hp
235	+	152276/153112	Putative transcriptional regulator [*Geobacillus* virus E3]	4e-18	HTH_24 (222-252)	0.01	Phage_rep_O ; Bacteriophage replication protein O 97.02/0.00032 phage_O_Nterm; phage replication protein O, N-terminal domain. 96.81/0.00082	Putative lambda O-type replication initiator

236	-	153575/153345	hp					hp
237	-	(154702/153686)	Site-specific tyrosine recombinase [*Bacillus* phage pW2]	0.0	recomb_XerD (34-193)	$3.22e^{-8}$		Xer family site-specific tyrosine recombinase, putative XerD recombinase
					Phage_integrase (130-281)	$1.24e^{-7}$		
					DNA_BRE_C (134-286)	$3.48e^{-5}$		
					INT_RitC_C_like (131-193)	$8.37e^{-5}$		
					Phage_int_SAM_1 (34-106)	$4.91e^{-3}$		
238	-	(155780/154923)	Plasmid replication-relaxation protein [*Bacillus* phage CampHawk]	$1e^{-21}$	Replic_Relax (179-242)	$2.03e^{-7}$	Replic_Relax ; Replication-relaxation 99.56/$1.5e^{-17}$	Replic_Relax superfamily protein
239	+	155855/156388	NUMOD4 motif protein [*Bacillus* phage vB_BcoS-136]	$6e^{-59}$	HNH_3 (66-110)	$3.48e^{-10}$	4.1.3 (M:1-105) Intron-encoded homing endonuclease I-HmuI {Bacteriophage SPO1 [TaxId: 10685]} 99.91/$1.4e^{-26}$	HNH homing endonuclease
					NUMOD4 (2-57)	$5.82e^{-7}$		
240	-	159184/156392	p-loop containing nucleoside triphosphate hydrolase [*Bacillus* phage pW2]	0.0			Bac_Flav_CT_G; Bacteroides conjugation system ATPase, TraG family 99.91/$1.3e^{-23}$ conj_TOL_TraD; conjugative coupling factor TraD, TOL family 99.91/$5.4e^{-25}$ TraG-Ti; Ti-type conjugative transfer system protein TraG 99.9/$8e^{-25}$ Bacterial conjugative coupling protein	hp

							TrwB {Escherichia coli 99.9/3.8e^{-25}	
241	-	159336/159184	hp					hp
242	-	160083/159361	M23 peptidase domain containing protein [*Bacillus* phage pW2]	2e^{-160}	Peptidase_M23 (28-125)<br>NlpD (8-149)<br>PRK11649 (11-112)	1.28e^{-18}<br>1.75e^{-18}<br>1.10e^{-9}	NlpD; Murein DD-endopeptidase MepM and murein hydrolase activator NlpD, contain LysM domain [Cell wall/membrane/envelope biogenesis]. 99.42/3.2e^{-14}	Endopeptidase
243	-	160578/160096	hp					hp
244	+	160697/161005	HNH homing endonuclease [*Staphylococcus* phage vB_Sau_Clo6]	1e^{-26}	NUMOD4 (2-57)	6.04e^{-14}	d.4.1.3 (M:1-105) Intron-encoded homing endonuclease I-HmuI {Bacteriophage SPO1 [TaxId: 10685]} 99.86/5.9e^{-24}	Homing endonuclease
245	+		HNH homing endonuclease [*Salmonella* phage vB_SenS_SB13	4e^{-7}				hp
246	-	161024/161230	hp				ParG 92.17/0.56 Transcriptional repressor CopG 91.27/0.79 Arc repressor {*Salmonella* bacteriophage P22 90.36/0.78 Mnt repressor {*Salmonella* bacteriophage P22 87.55/2.1	Putative ParG-like protein

247	-	161820/161536	hp		ParM_like (6-267)	$1.66e^{-21}$		ParM-like protein
248	+	162662/161832<br>162896/165214	Putative helicase-like protein [*Bacillus* phage pW2]	0.0	RecD (106-742)<br>DEXSc_RecD-like (352-479)<br>AAA_30 (352-497)	$2.69e^{-70}$<br>$2.30e^{-35}$<br>$1.68e^{-30}$		RecD-like DNA helicase
249	+	165331/166164	hp					hp
250	+	166229/166498	hp					hp
251	+	166550/166915	hp		Phage_gp49_66 (26-77)	$2.18e^{-13}$		Phage_gp49_66 superfamily protein
252	+	166926/167129	hp					hp
253	+	167135/167347	hp					hp
254	+	167349/167543	hp					hp
255	+	167543/167794	hp				Com; Mu-like prophage FluMu protein Com 97.91/$2.4e^{-7}$<br>A2L_zn_ribbon; 97.83/$3.4e^{-7}$	hp
256	+	167799/167996	hp					hp

* hp - hypothetical protein
